# Effect of dietary near ideal amino acid profile on heat production of lactating sows exposed to thermal neutral and heat stress conditions

**DOI:** 10.1186/s40104-020-00483-w

**Published:** 2020-07-09

**Authors:** S. Zhang, J. S. Johnson, N. L. Trottier

**Affiliations:** 1 Department of Animal Science, Michigan State University, East Lansing, 48824 USA; 2 USDA-ARS Livestock Behavior Research Unit, West Lafayette, 47907 USA

**Keywords:** Amino acid, Heat stress, Lactating sows, Low protein diet, Total heat production

## Abstract

**Background:**

Reduced protein diet manifested potential to mitigate heat production based on the concept of ideal amino acid profile. The hypothesis of this study was that lactating sows fed a low crude protein (LCP) diet with supplemental amino acid produce less heat compared to those fed a high crude protein (HCP) diet under both thermal neutral (TN) and heat stress (HS) conditions.

**Methods:**

Thirty-two lactating sows were allotted to HCP (193 g CP/kg) and LCP (140 g CP/kg) diets under thermal neutral (TN, 21 ± 1.5 °C) or cycling heat stress (HS, 32 ± 1.5 °C daytime and 24 ± 1.5 °C nighttime) conditions. Diets contained 0.90% SID lysine and 10.8 MJ/kg net energy. Positive pressure indirect calorimeters were used to measure gas exchange in individual sows with litters, and individual piglets on days 4, 8, 14 and 18. Sow and litter weights were recorded on days 1, 10 and 21.

**Results:**

Sow total heat production (THP) was calculated by subtracting litter THP from sow + litter THP based on BW^0.75^. Sow BW and body protein (BP) loss was greater for LCP diet compared to HCP diet in peak lactation (*P* < 0.05 and *P* < 0.01, respectively) and throughout the entire lactation period (*P* < 0.05 and *P* = 0.056, respectively) under HS conditions. Heat-stressed sows fed HCP diet had higher (*P* < 0.05) rectal temperature at 13:00 (*P* < 0.05) and 19:00 (*P* < 0.01), and higher respiration rate at 07:00 (*P* <  0.05), 13:00 (*P* < 0.05) and 19:00 (*P* < 0.05) compared to TN sows fed HCP diet. In sows fed LCP diet, those under HS tended to have higher (*P* = 0.098) rectal temperature at 13:00 and had higher (*P* <  0.05) respiration rate at 07:00, 13:00 and 19:00 compared to TN sows. The relationship between daily THP and days in lactation of sows fed LCP diet was quadratic (*P* < 0.05), with an ascending trend until day 14 and a descending trend from days 14 to 18. Sows fed LCP diet had lower daily THP at day 18 (*P* < 0.001) compared to those fed the HCP diet under HS conditions.

**Conclusion:**

Reduction in THP in sows fed LCP diet was largely associated with THP on day 18 of lactation under HS conditions. Feeding LCP diets alleviated the increased body temperature in sows under HS conditions throughout lactation, which was accompanied by a reduction in respiration rate. Total heat production is associated with days in lactation, in particular under HS conditions with THP appearing to peak between days 14 and 18.

## Background

Despite various cooling strategies, swine production systems are suboptimal in the summer [1]. Heat stress (HS) causes a series of adaptive behavioral and metabolic changes [2], including reduced voluntary feed intake [3, 4] and milk production in sows [5], elevated respiration rate (RR) and body temperature [6], and increased lipid tissue deposition in growing pigs [7, 8]. Swine are naturally HS sensitive due to a lack of functional sweat glands [9] and the existence of a substantial subcutaneous fat layer [8]. Newer genetic lines for greater lean yield have also contributed to an increase in metabolic heat production [7, 10]. In 2003, heat stress contributed to $360 million in annual economic losses to the United States swine industry [1]. This figure increased to $900 million in 2010 [11].

Greater metabolic rate during lactation due to the intense demand for milk production and litter-rearing [12] increases heat sensitivity [5] and HS risk to a larger extent than other production stages [4]. Therefore, reducing heat production in lactating sows exposed to high environmental temperatures may improve production efficiency and welfare. Reducing dietary protein decreases metabolic heat production in growing-finishing pigs [13, 14]. It was estimated [15] that heat production at peak lactation decreased from 288.7 to 154.8 MJ/(d·BW^0.75^) in sows housed under thermal neutral (TN) conditions by lowering dietary CP from 187 to 138 g/kg. The study objective was to use indirect calorimetry to measure heat production of individual lactating sows fed a diet containing either 184 g CP/kg or a NIAA diet containing 136 g CP/kg and housed under TN or HS environments. It was hypothesized that feeding a reduced-CP diet formulated to contain a near ideal amino acid (NIAA) profile decreases total metabolic heat production in lactating sows under TN and HS conditions compared to feeding a non-reduced CP diet formulated to meet SID lysine requirement with feed ingredients as sole sources of lysine.

## Methods

### Animals, feeding and experimental design

The experiment was conducted at the USDA-ARS Livestock Behavior Research Unit (West Lafayette, IN) in four consecutive blocks. Thirty-two multiparous (parity 3.25 ± 0.54) lactating Yorkshire × Landrace sows were used, with 8 sows randomly assigned to 1 of 2 dietary treatments per block. In each block, sows were individually housed in farrowing stalls, with 6 located in chambers [12], and 2 for backup substitutes outside of chambers. Sows were exposed to a TN environment (21.0 ± 1.5 °C and 41.8% ± 6.5% relative humidity) in blocks 2 and 4, or cycling HS environment (24.0 ± 1.7 °C and 32.0 ± 1.3 °C during nighttime and daytime, respectively, and 47.3% ± 5.4% relative humidity) in blocks 1 and 3, described in further details below. All sows were acclimated to diets (2.2 kg/d) and ambient temperature 6 days prior to farrowing. After farrowing, HS sows in blocks 1 and 3 were provided *ad libitum* access to feed. Feed allowance of TN sows (i.e., blocks 2 and 4) was calculated based on feed intake of HS sows within the respective dietary treatments from the preceding block including the backup substitute sows. Feed was provided 3 times daily, and orts were weighed and discarded every other day to avoid interfering with calorimetry day and maintain protocol consistency. No creep feed was provided to piglets and all animals had free access to water. Tail docking, ear notching, teeth clipping, iron injection, and castration were performed according to farm protocol 24 h post birth. Sows were housed in farrowing crates, and litters were standardized to 11.5 ± 0.9 piglets within the first 24 h of birth. Sow and litter weights were recorded, and sow backfat was measured with a backfat scanner (Lean-meater®, series 12, Renco Corp., Golden Valley, MN, USA) on days 1, 10, and at weaning. Milk samples were obtained from all sows on days 6 and 16 to represent early and peak lactation, respectively. Piglets were weaned on day 18 ± 1.7 due to farrowing schedule and constraints of the breeding program. Two sows were weaned on days 15 and 16 and their performance data (feed intake, litter weight gain, piglet ADG) from day 10 to weaning were excluded from the analyses.

### Dietary treatments

Ingredients and calculated nutrient composition of the diets are presented in Table 1. Analyzed total (hydrolysate) and free AA concentrations are presented in Table 2. The NRC model [16] was used to estimate requirements for AA, net energy (NE), calcium (Ca) and phosphorus (P). The requirements were predicted based on the following parameters: sow BW of 210 kg, parity number of 2 and above, and daily intake of 6 kg/d, litter size of 10, and piglet ADG of 280 g/d over a 21-day lactation period. The model predicted a minimum sow BW loss of 7.5 kg and the protein:lipid in the model was adjusted to the minimum allowable value of near zero. All diets were formulated to contain the same SID lysine (0.90%) and NE (10.8 MJ/kg) concentrations.

**Table 1:** Ingredient composition and nutrient content of experimental diets (g/kg, as-fed basis)

	High crude protein	Low crude protein
Ingredient composition		
Corn, yellow dent	591.7	614.5
Soybean meal^a^	300.0	140.0
Soy hulls	–	105.7
Sugar food product^b^	50.0	50.0
Beef tallow	33.5	50.2
*L*-Lysine·HCl	–	4.7
*L*-Valine	–	2.9
L-Threonine	–	2.0
*L*-Phenylalanine	–	1.3
*DL*-Methionine	–	1.1
*L*-Isoleucine	–	0.8
*L*-Histidine	–	0.7
*L*-Tryptophan	–	0.5
*L*-Leucine	–	–
Limestone	11.8	9.3
Dicalcium phosphate	4.5	7.8
Sodium chloride	5.0	5.0
Vitamin and mineral premix^c^	2.5	2.5
Titanium dioxide	1.0	1.0
Calculated nutrient^d^		
Net energy, MJ/kg	10.8	10.8
Crude protein	192.4	140
Fermentable fiber	115.8	115.8
SID^e^ amino acids		
Arginine	11.7	7.1
Histidine	4.7	3.7
Isoleucine	7.1	5.2
Leucine	14.7	10.3
Lysine	9.0	9.0
Methionine^f^	2.7	3.0
Methionine + cysteine	5.4	4.9
Phenylalanine	8.4	6.7
Phenylalanine + tyrosine	13.8	10.3
Threonine	6.1	5.8
Tryptophan	2.1	1.7
Valine	7.7	7.9
Nitrogen	26.3	18.8
Total calcium^g^	6.5	6.5
STTD^h^ phosphorus^g^	2.3	2.3

^a^480 g/kg crude protein

^b^Supplied per kg: net energy 11.9 MJ; fermentable fiber 0.5 g/kg; crude protein 10 g/kg (International Ingredient Corporation, St. Louis, MO, USA)

^c^Sow micro 5 and Se-yeast PIDX15 (Provimi North America, Inc. Brookville, Ohio, USA)

^d^Based on nutrient concentrations in feed ingredients [16]

^e^*SID* standardized ileal digestibility coefficient [16]

^f^Methionine concentration in Optimal and Optimal + Leucine is higher than Control because methionine was added to meet requirement of (methionine + cysteine)

^g^Concentration of calcium and phosphorus were based on phytase activity from the premix

^h^*STTD* standard total tract digestibility coefficient

**Table 2:** Analyzed and calculated concentration of nitrogen, total and free amino acids in high crude protein (HCP) and low crude protein (LCP) diets^a^ (g/kg, as-fed basis)

Item	HCP		LCP	
	Analyzed	Calculated^b^	Analyzed	Calculated^b^
Total				
Dry matter	887.6	–	889.5	–
Nitrogen	30.0	30.8	22.0	22.4
Arginine	12.3	12.6	7.5	7.8
Histidine	4.9	5.3	3.9	4.3
Isoleucine	8.5	8.1	6.1	6.0
Leucine	16.5	16.7	11.4	11.9
Lysine	11.1	10.4	10.8	10.1
Methionine	2.7	3.1	2.7	3.3
Methionine + cysteine	5.6	6.3	4.8	5.7
Phenylalanine	9.8	9.6	7.5	7.6
Phenylalanine + tyrosine	16.0	15.9	11.9	12.0
Threonine	7.2	7.3	6.4	6.8
Tryptophan	2.5	2.3	1.8	1.9
Valine	9.4	9.0	8.9	8.9
Free amino acids				
Arginine	0.3	–	0.1	–
Histidine	–	–	0.7	0.7
Isoleucine	0.1	–	0.8	0.8
Leucine	0.1	–	0.1	–
Lysine	0.2	–	3.6	3.7
Methionine^c^	–	–	0.7	1.1
Methionine + cysteine	–	–	0.7	1.1
Phenylalanine	–	–	1.2	1.3
Phenylalanine + tyrosine	0.1	–	1.2	1.3
Threonine	0.2	–	2.0	2.0
Tryptophan^d^	–	–	–	0.5
Valine	–	–	2.7	2.9

^a^Analyzed values represents average across 3 blocks (feed mixes)

^b^Calculated values for the total amino acids are based on the amino acids concentration in feed ingredients [16], and calculated values for the free amino acids correspond to the dietary inclusion rate in crystalline form

^c^Addition of *DL*-Methionine was omitted in one of the 3 blocks, thus reducing the overall free methionine concentration across all 3 blocks. The average free methionine concentration between blocks 1 and 3 was 0.11 and was zero in block 2. Therefore, across blocks 1, 2 and 3, average free Met was 0.07

^d^Analysis of free tryptophan was not performed

The control diet was formulated using corn and soybean meal as the only sources of lysine to meet NRC [16] SID lysine requirement (0.90%) and consequently contained 187.5 g CP/kg. Valine SID concentration was 0.77% which was near SID requirement of 0.79% [16]. All other indispensable amino acid (IDAA) SID concentrations were in excess relative to NRC [16]. This diet is referred to as the high crude protein (HCP) throughout the remainder of this manuscript.

A second diet balanced to reach a near ideal AA (NIAA) profile was formulated as described in Zhang et al. [17]. Briefly, the NIAA diet was designed by reducing soybean meal relative to corn to meet the minimum SID leucine requirement 1.03%, which corresponded to a CP concentration of 137.5 g/kg. Then, supplemental crystalline source of l-histidine, l-isoleucine, l-lysine, dl-methionine, *L*-histidine, *L*-isoleucine, *L*-lysine, *DL*-methionine, *L*-phenylalanine, *L*-threonine, *L*-tryptophan and *L*-valine were added to meet the minimum SID requirement for those AA. Crystalline dl-methionine was added to meet the requirement of methionine + cysteine. This diet is referred to as the low crude protein (LCP) diet throughout the remainder of the manuscript.

### Environmental control and physiological monitoring

Under TN conditions, ambient temperature was kept constant at 21.0 ± 1.5 °C, beginning 6 days prior to expected farrowing through weaning. Under HS conditions, a cycling HS approach was used to simulate natural temperature variations over a 24-h period during the summer season. Sows were progressively adapted to increasing ambient temperature over a 6-day period prior to the expected farrowing date, with the basal room temperature of 21.0 °C increased by 1.8 °C per day to a maximum of 32 °C by day 7, which corresponded to day 114 of gestation. The nighttime temperature under HS conditions was maintained at 24 °C. By day 2, the temperature exceeded 24 °C, therefore it was gradually decreased beginning at 15:00 to reach 24 °C by 19:00. During lactation, the room temperature was gradually increased every day from 24.0 °C beginning at 07:00 to 32.0 °C at 11:00, and thereafter the ambient temperature was maintained at 32.0 °C until 15:00. The temperature was gradually decreased beginning at 15:00 to reach 24.0 °C by 19:00.

Physiological indicators of HS included body temperature (vaginal and rectal temperature) and RR. Vaginal temperature was recorded in 10 min intervals, 24 h per day starting at day 3 of lactation until weaning using vaginal implants (iButton, accuracy ±0.1 °C; Dallas Semi-conductor, Maxim, Irving, TX) as previously described [18, 19]. Rectal temperature and RR were recorded daily at 07:00, 13:00, and 19:00 starting at lactation day 1 until day of weaning. Respiration rate (breaths/min) was measured by counting flank movement for 15 s and multiplying by 4 as previously described [19]. Lights were automatically turned off and on at 21:00 and 06:00, respectively.

### Indirect Calorimetry

In each block, six sows and their litters were housed in indirect calorimetry chambers and THP was determined on days 4–5, 8, 14–15 and 16–19 of lactation (corresponding to days 4, 8, 14 and 18, respectively, in the remainder of the manuscript). One sow (LCP, block 2, TN) farrowed later than her expected due date and therefore did not participate in the last calorimetry measurement day (i.e., day 18) due to constraints of the breeding schedule. Another sow (LCP, block 1, HS) completed half of her last calorimetry day on day 16 also due to her late farrowing date relative to her expected day. These 2 sows were weaned on days 15 and 16, respectively. Calorimetry was conducted in accordance with methods described in detail by Johnson et al. [12]. Briefly, within each indirect calorimetry testing day, THP was determined from 19:00–07:00 (overnight), 07:00 (pre-feeding), 08:00, 09:00, 10:00, 11:00, 13:00 (pre-feeding), 15:00 and 19:00 (pre-feeding). Indirect calorimetry was also conducted on sentinel piglets to measure their THP on days 4, 8, 14 and 18, and detailed in Johnson et al. [12]. The sentinel litter data were then used as a correction factor to estimate THP of the individual test sows as previously described [12].

### Nutrient analysis for diet and Milk

Feed was subsampled and submitted to the Agricultural Experiment Station Chemical Laboratories (University of Missouri-Columbia, Columbia, MO, USA) for AA analysis as previously described [17] to verify accuracy of feed mixing. Milk samples were submitted to the Michigan Dairy Herd Improvement Association (NorthStar Cooperative, Lansing, MI, USA) for analyses of fat, true protein, lactose, total solids and milk urea N (MUN) using infrared spectroscopy.

### Calculations

#### *Milk N concentration*

Milk N concentration was calculated [16] based on milk true protein and milk MUN concentrations as follows (Eq. 1):

1 $$ \mathrm{Milk}\ \mathrm{N}\ \mathrm{concentration}\ \left(\%\right)=\mathrm{milk}\ \mathrm{true}\ \mathrm{protein}\ \left(\%\right)\times 6.38+\mathrm{MUN}\ \left(\%\right) $$

#### *Milk energy concentration*

The milk energy content was calculated [20] as follows (Eq. 2):

2 $$ \mathrm{Milk}\ \mathrm{energy}\ \left(\mathrm{kJ}/\mathrm{g}\right)=\mathrm{fat}\%\times 39.7+\mathrm{protein}\%\times 23.8+\mathrm{lactose}\%\times 16.5 $$

#### *Heat production*

Heat production was calculated [21] as follows (Eq. 3):

3 $$ \mathrm{Heat}\ \mathrm{production}\ \left(\mathrm{kJ}\right)=16.2\times {\mathrm{O}}_2\ \left(\mathrm{L}\right)+5.0\times {\mathrm{CO}}_2\left(\mathrm{L}\right) $$

Urinary N excretion accounts for only 0.24–0.64% of the THP in pigs [22], therefore it was not included in the calculation.

Sow metabolic CO_2_ (Eq. 4), O_2_ (Eq. 5) and THP (Eq. 6) was calculated by subtracting litter THP from sow + litter THP based on BW^0.75^ of sow and litter, respectively.

4 $$ \mathrm{Sow}\ \mathrm{metabolic}\ {\mathrm{CO}}_2\left(\mathrm{L}\bullet {\mathrm{d}}^{-1}\bullet {\mathrm{BW}}^{-0.75}\right)=\frac{\mathrm{Sow}\ \mathrm{and}\ \mathrm{litter}\ {\mathrm{CO}}_2\ \left(\mathrm{L}/\mathrm{d}\right)-\mathrm{litter}\ \mathrm{metabolic}\ {\mathrm{CO}}_2\ \left(\mathrm{L}\bullet {\mathrm{d}}^{-1}\bullet {\mathrm{BW}}^{0.75}\right)\times {\mathrm{LW}}^{0.75}}{\mathrm{Sow}\ {\mathrm{BW}}^{0.75}} $$

5 $$ \mathrm{Sow}\ \mathrm{metabolic}\ {\mathrm{O}}_2\left(\mathrm{L}\bullet {\mathrm{d}}^{-1}\bullet {\mathrm{BW}}^{-0.75}\right)=\frac{\mathrm{Sow}\ \mathrm{and}\ \mathrm{litter}\ {\mathrm{O}}_2\ \left(\mathrm{L}/\mathrm{d}\right)-\mathrm{litter}\ \mathrm{metabolic}\ {\mathrm{O}}_2\ \left(\mathrm{L}\bullet {\mathrm{d}}^{-1}\bullet {\mathrm{BW}}^{0.75}\right)\times {\mathrm{LW}}^{0.75}}{\mathrm{Sow}\ {\mathrm{BW}}^{0.75}} $$

6 $$ \mathrm{Sow}\ \mathrm{metabolic}\ \mathrm{THP}\ \left(\mathrm{kJ}\bullet {\mathrm{d}}^{-1}\bullet {\mathrm{BW}}^{0.75}\right)=\frac{\mathrm{Sow}\ \mathrm{and}\ \mathrm{litter}\ \mathrm{THP}\ \left(\mathrm{kJ}/\mathrm{d}\right)-\mathrm{litter}\ \mathrm{metabolic}\ \mathrm{THP}\ \left(\mathrm{kJ}\bullet {\mathrm{d}}^{-1}\bullet {\mathrm{BW}}^{0.75}\right)\times {\mathrm{LW}}^{0.75}}{\mathrm{Sow}\ {\mathrm{BW}}^{0.75}} $$

Litter weight (LW) could not be recorded on calorimetry days (days 4, 8, 14 and 18), therefore LW was estimated by assuming linear growth rate from days 1 to d 10 and from days 10 to wean day (Eq. 7, 8, 9, 10).

7 $$ {\mathrm{LW}}_{\mathrm{d}4}\ \left(\mathrm{kg}\right)={\mathrm{LW}}_{\mathrm{d}1}\left(\mathrm{kg}\right)+\frac{{\mathrm{LW}}_{\mathrm{d}10}\left(\mathrm{kg}\right)-{\mathrm{LW}}_{\mathrm{d}1}\left(\mathrm{kg}\right)}{\mathrm{d}10-\mathrm{d}1}\times \left(\mathrm{d}4-\mathrm{d}1\right) $$

8 $$ {\mathrm{LW}}_{\mathrm{d}8}\ \left(\mathrm{kg}\right)={\mathrm{LW}}_{\mathrm{d}1}\left(\mathrm{kg}\right)+\frac{{\mathrm{LW}}_{\mathrm{d}10}\left(\mathrm{kg}\right)-{\mathrm{LW}}_{\mathrm{d}1}\left(\mathrm{kg}\right)}{\mathrm{d}10-\mathrm{d}1}\times \left(\mathrm{d}8-\mathrm{d}1\right) $$

9 $$ {\mathrm{LW}}_{\mathrm{d}14}\left(\mathrm{kg}\right)={\mathrm{LW}}_{\mathrm{d}10}\left(\mathrm{kg}\right)+\frac{{\mathrm{LW}}_{\mathrm{wean}}\left(\mathrm{kg}\right)-{\mathrm{LW}}_{\mathrm{d}10}\left(\mathrm{kg}\right)}{{\mathrm{d}}_{\mathrm{wean}}-\mathrm{d}10}\times \left(\mathrm{d}14-\mathrm{d}10\right) $$

10 $$ {\mathrm{LW}}_{{\mathrm{d}}_{\mathrm{wean}}}\left(\mathrm{kg}\right)={\mathrm{LW}}_{\mathrm{d}10}\ \left(\mathrm{kg}\right)+\frac{{\mathrm{LW}}_{\mathrm{wean}}\ \left(\mathrm{kg}\right)-{\mathrm{LW}}_{\mathrm{d}10}\left(\mathrm{kg}\right)}{{\mathrm{d}}_{\mathrm{wean}}-\mathrm{d}10}\times \left({\mathrm{d}}_{\mathrm{wean}}-\mathrm{d}10\right) $$

### Statistical analysis

Data were analyzed by ANOVA using the Mixed model procedures of SAS 9.4 (SAS Inst. Inc., Cary, NC). For the analysis of performance (Table 3), body composition (Table 4) and milk composition (Table 5) data, the following model was used:

**Table 3:** Performance of litter and sow fed high crude protein (HCP; 192.4 g/kg crude protein) and low crude protein (LCP; 140.0 g/kg crude protein) diets and exposed to thermal neutral and heat stress conditions^a^

Item	Thermal neutral				Heat stress			
	HCP	LCP	SEM^b^	*P*-value	HCP	LCP	SEM^b^	*P*-value
Number of sows^c^	6	6	–	–	6	6	–	–
Parity	3	3	–	–	3	4	–	–
Wean day	19	18	–	–	19	17	–	–
Sow ADFI^d^, kg/d								
Overall	6.47	6.01	0.24	0.295	6.47	5.88	0.24	0.185
Early	5.66	5.20	0.24	0.308	5.73	5.43	0.24	0.505
Peak	7.36	6.93	0.24	0.347	7.36	6.83	0.24	0.252
Sow BW, kg								
Day 1	217.7	214.0	15.0	0.869	222.0	249.8	15.0	0.220
Day 10	220.2	211.4	13.7	0.669	223.7	247.5	13.7	0.253
Wean	209.7	206.7	14.5	0.878	221.4	237.2	14.5	0.422
Sow BW change^d^, g/d								
Overall	− 433.6	− 426.8	188.5	0.982	−35.6	−790.6	188.5	0.023
Early	120.0	−154.6	128.6	0.177	83.9	− 142.8	128.6	0.262
Peak	− 553.7	− 272.2	128.6	0.167	− 119.5^*^	− 647.8^†^	128.6	0.014
Sow back fat, mm								
Day 1	14.4	14.5	2.2	0.974	15.0	15.5	2.2	0.834
Day 10	13.7	13.4	1.8	0.892	14.7	14.6	1.8	0.964
Wean	12.6	11.3	2.0	0.496	13.5	13.8	2.0	0.898
Sow back fat change, mm/d								
Overall	−0.100	−0.191	0.038	0.246	−0.077	−0.102	0.038	0.749
Early	−0.043	−0.074	0.039	0.625	−0.015	−0.055	0.039	0.519
Peak	−0.057	−0.118	0.039	0.336	−0.062	−0.047	0.039	0.803
Litter size								
Day 1	12	11	–	–	12	11	–	–
Day 10	11	11	–	–	11	11	–	–
Wean	11	11	–	–	11	10	–	–
Piglet daily gain, g/d								
Overall	259.7	255.2	34.3	0.849	220.3	230.0	34.3	0.683
Early	251.6	249.0	33.9	0.931	216.7	245.8	33.9	0.341
Peak	268.8	268.2	33.9	0.985	232.2	231.2	33.9	0.975
Litter weight gain, kg/d								
Overall	2.94	2.81	0.29	0.650	2.49	2.37	0.29	0.663
Early	2.91	2.74	0.29	0.631	2.56	2.57	0.29	0.970
Peak	2.98	2.96	0.29	0.962	2.49	2.35	0.29	0.686

^a^Data are least squares means. Overall: d 1-wean; early: d 1–10; peak: d 10-wean

^b^Maximum value of the standard error of the means

^c^Two sows were weaned on days 15 (LCP under TN) and 16 (LCP under HS) and their performance data (feed intake, litter weight gain, piglet ADG for day 10 to weaning) were excluded from the analyses

^d^The main effect of lactation stage (early vs. peak) was significant for sow body weight (BW) change and average daily feed intake (ADFI)

^*^Within the same diet, environments differed (*P* < 0.05)

^†^Within the same diet, environments tended to differ for BW change at peak lactation (*P* = 0.052)

**Table 4:** Body composition of sow fed high crude protein (HCP; 192.4 g/kg crude protein) and low crude protein (LCP; 140.0 g/kg crude protein) diets and exposed to thermal neutral and heat stress conditions^a^

Item	Thermal neutral				Heat stress			
	HCP	LCP	SEM^b^	*P*-value	HCP	LCP	SEM^b^	*P*-value
Number of sows^c^	6	6	–	–	6	6	–	–
Parity	3	3	–	–	3	4	–	–
Body protein, %								
Day 1	16.7	16.6	0.3	0.841	16.5	16.6	0.3	0.877
Day 10	16.8	16.7	0.3	0.849	16.6	16.7	0.3	0.639
Wean	16.9	17.1	0.3	0.565	16.7	16.8	0.3	0.863
Protein change^d^, g/d								
Overall	−38.7	−7.1	29.7	0.560	20.9	−87.5^†^	29.7	0.056
Early	74.8	−9.8	55.0	0.329	42.4	−10.4	55.0	0.540
Peak	−161.3	−22.2	55.0	0.116	2.2^*^	− 267.9^*^	55.0	0.005
Protein tissue change^d^, g/d								
Overall	−193.5	−35.5	148.5	0.560	104.5	− 437.5^†^	148.5	0.056
Early	374.0	−49	275.0	0.329	212.0	−52.0	275.0	0.540
Peak	− 806.5	− 111.0	275.0	0.116	11.0^*^	− 1339.5^*^	275.0	0.005
Body lipid, %								
Day 1	18.0	18.2	1.8	0.926	19.0	19.7	1.8	0.729
Day 10	17.8	17.7	1.5	0.925	18.9	19.1	1.5	0.897
Wean	16.8	15.9	1.7	0.572	18.1	18.4	1.7	0.845
Lipid change^d^, g/d								
Overall	− 206.2	− 337.9	64.1	0.296	− 105.3	− 276.7	64.1	0.179
Early	−52.5	− 222.1	137.2	0.438	−3.8	−190.6	137.2	0.394
Peak	− 415.9	−503.5	137.2	0.687	− 232.2	− 523.1	137.2	0.190
Lipid tissue change^d^, g/d								
Overall	− 247.4	− 405.5	76.9	0.296	− 126.4	− 332.0	76.9	0.179
Early	−63.0	− 266.5	164.6	0.438	−4.6	−228.7	164.6	0.394
Peak	− 499.1	− 604.2	164.6	0.687	− 278.6	− 627.7	164.6	0.190

^a^Data are least squares means. Overall: d 1-wean; early: d 1–10; peak: d 10-wean

^b^Maximum value of the standard error of the means

^c^Two sows were weaned on days 15 (LCP under TN) and 16 (LCP under HS) and their performance data (feed intake, litter weight gain, piglet ADG for day 10 to weaning) were excluded from the analyses

^d^The main effect of lactation stage (early vs. peak) was significant for sow body lipid (tissue) and body protein (tissue) change

^*^Within the same diet, environments differed (*P* < 0.05)

^†^Within the same diet, environments tended to differ for overall protein (tissue) change (*P* = 0.072)

**Table 5:** Milk yield and composition of sows fed high crude protein (HCP; 192.4 g/kg crude protein) and low crude protein (LCP; 140.0 g/kg crude protein) diets and exposed to thermal neutral and heat stress conditions^a^

Item	Thermal neutral				Heat stress			
	HCP	LCP	SEM^b^	*P*-value	HCP	LCP	SEM^b^	*P*-value
No. of sows	6	6	–	–	6	6	–	–
Early lactation^c^								
Yield, kg/d	9.1	9.1	1.4	0.987	7.8	9.0	1.4	0.480
True protein, %	4.04	3.89	0.13	0.532	4.10	3.71	0.13	0.105
Urea-N, mg/dL	12.95	3.93	1.89	< 0.001	11.05	2.13	1.89	< 0.001
N, %	0.646	0.614	0.020	0.381	0.653	0.583	0.020	0.063
Energy, kJ/g	461.1	491.6	26.4	0.257	439.7	500.8	26.4	0.032
Lactose, %	5.70	5.64	0.12	0.562	5.58	5.61	0.12	0.811
Fat, %	6.80	7.69	0.67	0.218	6.29	8.04	0.67	0.021
Peak lactation^c^								
Yield, kg/d	13.8	15.5	1.4	0.328	12.7	13.2	1.4	0.763
True protein, %	4.15	3.84	0.13	0.184	3.94	3.68	0.13	0.271
Urea-N, mg/dL	15.55	4.15	1.89	< 0.001	11.12	3.47	1.89	< 0.001
N, %	0.668	0.606	0.020	0.098	0.629	0.580	0.020	0.189
Energy, kJ/g	472.0	469.9	26.4	0.940	467.8	436.4	26.4	0.244
Lactose, %	5.82	5.86	0.12	0.728	5.62	5.66	0.12	0.732
Fat, %	6.95	7.09	0.67	0.851	7.07	6.41	0.67	0.358

^a^Data are least squares means

^b^Maximum value of the standard error of the means

^c^The main effect of lactation stage (early vs. peak) was significant for milk yield

Response = diet + environment + stage + *block*_*environment*_ + *sow*_diet × block_ + diet × environment + diet × stage + environment × stage + *e.*

The **response** of sow depended on the fixed effects of **diet (**HCP vs. LCP**)**, **environment** (TN vs. HS), and lactation **stage** (early vs. peak lactation, if applicable). The random effects included ***block*** nested within the environment (TN and HS), individual ***sow*** nested within diet and block. The interactive effects of **diet × environment**, **diet × stage**, and **environment × stage** were also included*.*

For the analysis of physiological data, rectal temperature and RR was first averaged over the lactation period for each sow at each measurement time (07:00, 13:00 and 19:00). (Table 6) and the following model was used:

**Table 6:** Thermoregulatory response of sows fed high crude protein (HCP; 192.4 g/kg crude protein) and low crude protein (LCP; 140.0 g/kg crude protein) diets and exposed to thermal neutral (TN) and heat stress (HS) conditions^a^

Item	HCP				LCP			
	TN	HS	SEM	*P*-value	TN	HS	SEM	*P*-value
No. of sows	6	6	–	–	6	6	–	–
Rectal body temp, °C								
07:00	38.93	39.11	0.16	0.427	38.99	39.02	0.16	0.906
13:00	39.23	39.82	0.16	0.012	39.28	39.65	0.16	0.098
19:00	39.32	40.03	0.16	0.003	39.33	39.68^*^	0.16	0.115
Respiration rate, #/min								
07:00	25	43	2	< 0.001	25	37^*^	2	< 0.001
13:00	30	76	2	< 0.001	30	74	2	< 0.001
19:00	28	55	2	< 0.001	29	51^†^	2	< 0.001

^a^Data are least squares means

^*^Diets differed within the same environment (*P* < 0.05)

^†^Diets tended to differ within the same environment (*P* = 0.085)

Response = diet + environment + time + *block*_*environment*_ + *sow*_diet × block_ + diet × environment + diet × time + environment × time + *e.*

The **response** of sow depended on the fixed effects of **diet (**HCP vs. LCP**)**, **environment** (TN vs. HS), and repeated measurements of **time** for body temperature and RR (07:00, 13:00 and 19:00). The random effect included ***block*** nested within the environment (TN and HS), individual ***sow*** nested within diet and block. The interactive effect of **diet × environment**, **diet × time**, and **environment × time** were also included*.*

**Fig. 1:**
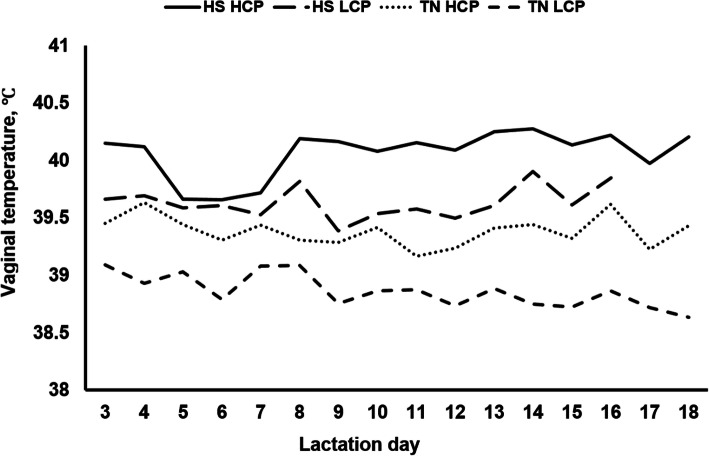
Vaginal temperature of sows fed high crude protein (HCP; 192.4 g/kg crude protein) and low crude protein (LCP; 140.0 g/kg crude protein) diets and exposed to thermal neutral (TN) and heat stress (HS) environments. Within the same environment (TN or HS), diets (LCP vs. HCP) differed (*P* < 0.01). Within the same diet (HCP or LCP), environments (HS vs. TN) differed (*P* < 0.01). Standard error of the mean, SEM = 0.183

For the analysis of vaginal temperature (Fig. 1), the following model was used:

Response = diet + environment + day + *block*_*environment*_ + *sow*_diet × block_ + diet × environment + diet × day + environment × day + *e.*

The vaginal temperature (i.e., **response**) was averaged daily, and depended on the fixed effects of **diet (**HCP vs. LCP**)**, **environment** (TN vs. HS), and repeated measurement of **day** of lactation. The random effects included ***block*** nested within the environment (TN and HS), individual ***sow*** nested within diet and block. The interactive effects of **diet × environment**, **diet × day**, and **environment × day** were also included*.*

The THP on days 4, 8, 14 and 18 of lactation was analyzed to compare dietary effect (HCP vs. LCP) within each environment (HS or TN) (Table 7). Under HS, ME intake (MEI) between diets varied, thus the MEI was included as a covariable in the model as follows:

**Table 7:** Feed intake and metabolic total heat production [kJ/(d·BW^0.75^)] of lactating sows fed high crude protein (HCP; 192.4 g/kg crude protein) and low crude protein (LCP; 140.0 g/kg crude protein) diets and exposed to thermal neutral and heat stress conditions^a^

Item	Thermal neutral				Heat stress			
	HCP	LCP	SEM^b^	*P*-value	HCP	LCP	SEM^b^	*P*-value
Number of sows^c^	6	6	–	–	6	6	–	–
Feed intake, kg/d^d^								
Day 4	4.96	4.46	–	–	5.23	4.83	0.46	0.536
Day 8	6.59	5.70	–	–	6.63	5.58	0.46	0.109
Day 14	6.93	6.57	–	–	6.97	6.56	0.46	0.524
Day 18^c^	7.26	7.80	–	–	7.58	6.93	0.46	0.406
Metabolic total heat production								
Nighttime (19:00–07:00)^e^								
Day 4	563.6	465.7	53.6	0.203	597.5	512.1	43.1	0.092
Day 8	568.2	533.0	53.6	0.645	589.9	538.9	41.4	0.263
Day 14	651.9	577.0	53.6	0.329	627.2	603.8	43.5	0.616
Day 18	612.1	493.7	58.6	0.145	573.6	457.3	41.0	0.013
Average	599.1	518.8	27.6	0.040	609.6	515.1	31.8	0.006
SEM^§b^	28.6	42.8			61.1	16.3		
Contrast^f^	–	–			–	Q^*^, D^†^		
Daytime (07:00–19:00)^e^								
Day 4	627.6	618.8	51.5	0.873	677.8	674.9	30.1	0.940
Day 8	729.7	682.8	51.5	0.393	692.5	658.6	28.5	0.410
Day 14	779.9	686.6	51.5	0.093	697.5	672.0	28.0	0.529
Day 18	724.7	664.8	54.4	0.301	711.3	544.8	28.0	< 0.001
Average	715.5	663.6	39.9	0.065	708.8	625.5	14.6	0.009
SEM^§b^	26.3	49.0			17.2	22.2		
Contrast^f^	L^*^, Q^*^, D^†^	–			–	L^*^, Q^*^, D^*^		
Overall 24 h								
Day 4	595.4	542.2	48.1	0.377	643.5	592.0	29.7	0.184
Day 8	648.9	607.9	48.1	0.494	640.2	597.5	28.5	0.259
Day 14	715.9	631.8	48.1	0.165	661.5	638.9	28.0	0.542
Day 18	668.6	579.9	51.9	0.164	641.4	500.0	28.0	< 0.001
Average	657.3	591.2	32.2	0.033	659.8	571.1	17.6	0.002
SEM^§b^	21.5	45.9			38.9	11.3		
Contrast^f^	–	–			–	Q^*^, D^*^		

^a^Data are least squares means

^b^Maximum value of the standard error of the means

^c^One LCP sow under TN was missing for calorimetry day 18 and one LCP sow under HS completed calorimetry day 18 from 07:00 until 12:00

^d^Feed intake under TN was fixed and pair fed to counterparts under HS, and thus no SEM and *P* value were included

^e^Metabolic total heat production between nighttime and daytime differs under TN and HS conditions (*P* < 0.01)

^f^Linear, quadratic contrast and day effect on total heat production along lactation (d 4, 8, 14 and 18) was performed and represented as L, Q, and D, respectively

^§^Standard error of the means for contrast over days 4, 8, 14 and 18

^*^ Within the same diet, environments differed (*P* < 0.05)

^†^ Within the same diet, environments tended to differ (0.05 < *P* ≤ 0.10)

Response = MEI + diet + day + *block* + *sow*_diet × block_ + diet × day + *e.*

The **response** of sow corrected for MEI depended on the fixed effects of **diet (**HCP vs. LCP**)** and repeated measurements of each calorimetry **day** (days 4, 8, 14 and 18). The random effects included ***block***, individual ***sow*** nested within diet and block. The interactive effect of diet × day was also included*.* Under TN, sows were pair-fed to HS counterparts, and therefore MEI was fixed. The MEI was not an independent and random variable, thus the model was the same as under HS except that the covariable MEI was not included.

The THP at different daytime points on days 4, 8, 14 and 18 of lactation was analyzed to compare dietary effect (HCP vs. LCP) within each environment (HS or TN) via double repeated measurements (day and sampling time) (Table 8). Under HS, MEI was included as a covariable in the model as follows:

**Table 8:** Metabolic total heat production [kJ/(d·BW^0.75^)] during daytime of lactating sows fed high crude protein (HCP; 192.4 g/kg crude protein) and low crude protein (LCP; 140.0 g/kg crude protein) diets and exposed to thermal neutral and heat stress conditions^a^

Item	Thermal neutral				Heat stress			
	HCP	LCP	SEM^b^	*P*-value	HCP	LCP	SEM^b^	*P*-value
Day 4								
07:00^c^	623.4	610.0	50.2	0.840	700.0	643.1	47.3	0.380
08:00	635.1	637.2	52.3	0.975	669.9	677.4	47.3	0.905
09:00	578.2	576.1	50.2	0.976	675.7	715.9	47.3	0.534
10:00	606.3	583.2	50.2	0.723	656.9	642.7	47.3	0.824
11:00	637.2	610.0	50.2	0.677	564.8	635.5	51.5	0.296
13:00	677.4	703.7	50.2	0.689	671.5	630.1	47.3	0.521
15:00	654.4	627.2	50.2	0.677	700.0	613.4	51.9	0.201
19:00	606.3	607.1	50.2	0.990	654.0	589.9	52.3	0.344
Day 8								
07:00^c^	672.4	605.8	60.7	0.419	690.8	569.0	44.8	0.061
08:00	748.9	726.3	60.7	0.784	743.9	679.5	44.8	0.317
09:00	683.7	613.0	60.7	0.389	715.9	667.8	44.8	0.454
10:00	733.9	668.2	60.7	0.422	673.6	612.5	44.8	0.345
11:00	721.7	699.6	60.7	0.785	692.5	662.7	44.8	0.648
13:00	754.8	724.7	60.7	0.716	706.3	603.3	44.8	0.113
15:00	792.4	742.7	60.7	0.544	779.5	658.1	44.8	0.062
19:00	728.9	682.4	60.7	0.570	655.2	711.3	44.8	0.381
Day 14								
07:00^c^	815.0	658.6	56.5	0.042	748.5	695.8	50.6	0.456
08:00	755.2	700.4	56.5	0.468	760.7	777.8	50.6	0.810
09:00	735.5	659.0	56.5	0.311	737.2	664.4	50.6	0.307
10:00	751.4	651.9	56.5	0.189	742.2	614.2	50.6	0.076
11:00	739.7	694.1	56.5	0.546	758.1	701.2	50.6	0.422
13:00	790.4	670.3	56.5	0.116	592.5	621.7	50.6	0.692
15:00	860.6	793.3	56.5	0.375	725.9	705.8	50.6	0.780
19:00	789.1	664.0	56.5	0.101	699.1	670.7	50.6	0.688
Day 18								
07:00^c^	742.7	668.6	71.1	0.430	736.4	619.7	52.3	0.118
08:00	802.9	694.1	71.1	0.246	765.3	552.3	52.3	0.005
09:00	672.4	630.9	71.1	0.659	657.7	527.2	52.3	0.080
10:00	638.5	551.0	71.1	0.350	649.4	454.4	52.3	0.010
11:00	678.6	634.7	71.1	0.640	724.7	501.2	52.3	0.004
13:00	734.3	659.0	71.1	0.421	849.8	554.8	56.9	0.001
15:00	803.3	723.0	71.1	0.391	785.3	531.4	56.9	0.006
19:00	725.1	623.8	71.1	0.279	686.2	584.1	56.9	0.227

^a^Data are least squares means

^b^Maximum value of the standard error of the means

^c^Total heat production before first morning meal

Response = MEI + diet + day + sampling time + *block* + *sow*_diet × block_ + diet × day + diet × sampling + day × sampling + *e.*

The **response** of sow was corrected by MEI and depended on the fixed effect of **diet (**HCP vs. LCP**)**, and double repeated measurements of calorimetry **day** (days 4, 8, 14 and 18) and **sampling time** (07:00, 08:00, 09:00, 10:00, 11:00, 13:00, 15:00 and 19:00) of CO_2_ and O_2_. The random effect included ***block***, individual ***sow*** nested within diet and block. The interactive effect of diet × day, diet × sampling time, and day × sampling time were also included*.* Under TN, the model was the same as under HS, except that the covariable was not included.

Effects were declared significant at *P* ≤ 0.05 and tendency were declared at 0.05 < *P* ≤ 0.10.

## Results

### Experimental diets

Diet composition and nutrient concentrations are presented in Table 1 and IDAA concentrations are presented in Table 2.

### Performance

Sow and litter performances are presented in Table 3.

#### *LCP vs. HCP*

Under TN and HS, daily feed intake and backfat loss, and litter weight gain did not differ between sows fed LCP and HCP diets at any stages of lactation. Under TN, BW loss did not differ between diets. Under HS, BW loss was greater for sows fed the LCP diet compared to the HCP diet in peak and entire lactation (*P* <  0.05) period.

#### *HS vs. TN*

For the HCP diet, daily feed intake, backfat loss, and litter weight gain did not differ between HS and TN exposed sows at any lactation stage. For the HCP diet, sows under HS conditions lost less BW (*P* <  0.05) in peak lactation, when compared to TN conditions. For the LCP diet, average daily feed intake, backfat loss, and litter weight gain did not differ between sows housed under HS or TN conditions at any lactation stage. For the LCP diet, sows under HS conditions tended to lose more BW compared to TN conditions (*P* = 0.052) in peak lactation.

### Body lipid and protein mobilization

Body lipid and protein mobilization data are presented in Table 4 and illustrated in Supplementary Figure 1.

#### *LCP vs. HCP*

Under TN conditions, body lipid (tissue) and body protein (tissue) mobilization did not differ between sows fed LCP and HCP diets at any lactation stages. Under HS conditions, body lipid (tissue) mobilization did not differ between sows fed LCP and HCP diets at any lactation stage. Under HS conditions, when compared to the HCP diet, sows fed the LCP diet mobilized and tended to mobilize more body protein (tissue) in peak (*P* <  0.01) and throughout the entire lactation phase (*P* = 0.056), respectively.

#### *HS vs. TN*

For the HCP diet, body lipid (tissue) mobilization did not differ between HS and TN conditions at any lactation stage. Sows fed the HCP diet under HS conditions mobilized less (*P* <  0.05) body protein (tissue) during peak lactation when compared to TN exposed sows. Body lipid (tissue) mobilization did not differ between HS and TN conditions for sows fed the LCP diet at any lactation stage. Sows fed the LCP diet under HS conditions mobilized more (*P* <  0.05) protein (tissue) in peak lactation and tended to lose more (*P* = 0.072) protein (tissue) in the entire lactation period compared to sows fed the LCP diet under TN conditions.

### Milk yield and composition

Milk composition data are presented in Table 5.

#### *LCP vs. HCP*

Under TN conditions, milk yield, milk true protein, lactose, fat and energy concentrations did not differ between sows fed the LCP and HCP diets at any lactation stage. Sows fed the LCP diet under TN conditions had lower MUN in both early (*P* < 0.01) and peak (*P* < 0.01) lactation and tended to have lower milk N concentration (*P* = 0.098) when compared to sows fed the HCP diet under TN conditions. Under HS conditions, milk yield, milk true protein and lactose did not differ between sows fed LCP and HCP diets at any lactation stage. Under HS conditions, when compared to the HCP diet, sows fed the LCP diet had lower MUN in both early (*P* < 0.01) and peak (*P* < 0.01) lactation, and higher milk energy (*P* < 0.05), fat (*P* < 0.05), and tendency for lower milk N concentration (*P* = 0.063) in early lactation. Under HS conditions, milk energy, fat, lactose and N concentrations did not differ between LCP and HCP fed sows in peak lactation.

#### *HS vs. TN*

No HS versus TN environmental differences were detected for milk production, milk true protein, MUN, N, energy, lactose and fat concentrations for sows fed either HCP or LCP diets.

### Physiological response to ambient temperature

The rectal temperature and RR data are presented in Table 6. Vaginal temperature data are depicted in Fig. 1.

#### *HS vs. TN*

Sows fed the HCP diet under HS conditions had higher rectal temperatures at 13:00 (*P* <  0.05) and 19:00 (*P* < 0.01), and higher RR at 07:00 (*P* < 0.01), 13:00 (*P* < 0.01) and 19:00 (*P* < 0.01) compared to sows fed the HCP diet under TN conditions. Under HS conditions, sows fed the LCP diet tended to have higher (*P* = 0.098) rectal temperatures at 13:00, and greater RR at 07:00 (*P* < 0.01), 13:00 (*P* < 0.01) and 19:00 (*P* < 0.01) when compared to sows fed the LCP diet under TN conditions. Overall, sows under HS conditions had higher (*P* < 0.01) vaginal temperatures throughout the entire lactation period when compared to TN exposed sows, regardless of dietary treatment.

#### *LCP vs. HCP*

Under TN, sow rectal temperature and RR did not differ between LCP and HCP diets at 07:00, 13:00 and 19:00. Under HS, compared to HCP diet, sows fed LCP diet had lower rectal temperature (*P* < 0.05) at 19:00, lower RR at 07:00 (*P* < 0.05) and tended to have lower RR at 19:00 (*P* = 0.085). Overall, sows fed the LCP diet had lower (*P* < 0.01) vaginal temperatures over the entire lactation period when compared to sows fed the HCP diet, regardless of environmental exposure.

### Heat production

Total heat production data are presented in Tables 7 and 8.

#### *Nighttime (19:00–07:00)*

Under HS conditions, sows fed the LCP diet tended to have lower THP at day 4 (*P* = 0.092), and lower THP at day 18 (*P* < 0.05) when compared to sows fed the HCP diet. No other THP differences were detected during nighttime for any comparison.

#### *Daytime (07:00–19:00)*

Under TN conditions, sows fed the LCP diet tended to have lower THP (*P* = 0.093) at day 14 when compared to sows fed the HCP diet. Under HS conditions, sows fed the LCP diet had lower THP (*P* < 0.01) at day 18 when compared to sows fed the HCP diet. No other THP differences were detected in daytime for any comparison.

#### *24-h period*

Under HS conditions, sows fed the LCP diet had lower THP on day 18 (*P* < 0.001) when compared to sows fed the HCP diet.

#### *Entire lactation period*

The relationship between daily (overall 24 h) THP of sows fed LCP diet as lactation progressed was quadratic (*P* < 0.05) under HS, showing an ascending trend until day 14 and a descending trend from days 14 to 18. This relationship was also observed for sows fed LCP diet under HS environment in daytime (07:00–19:00) (*P* < 0.05) and nighttime (19:00–07:00) (*P* < 0.05). For sows fed HCP diet, this relationship was quadratic under TN during daytime (07:00–19:00) (*P* < 0.05). There was no relationship between THP and days in lactation for sows fed HCP in nighttime under TN.

#### *Entire lactation period (daytime)*

Under TN conditions, sows fed the LCP diet had lower (*P* < 0.05) THP at 07:00 on day 14 when compared to sows fed the HCP diet. Under HS conditions, sows fed the LCP diet tended to have lower THP at 07:00 (*P* = 0.061) and 15:00 (*P* = 0.062) on day 8 when compared to sows fed the HCP diet. On day 14, THP tended to be lower in sows fed LCP diet than HCP diet at 10:00 (*P* = 0.076) and did not differ at other time points. On day 18, THP was lower at 08:00 (*P* < 0.01), 09:00 (*P* = 0.08), 10:00 (*P* < 0.01), 11:00 (*P* < 0.01), 13:00 (*P* < 0.01) and 15:00 (*P* < 0.01), and did not differ at 19:00 (Table 8).

## Discussion

Daily metabolic O_2_ consumption and CO_2_ production values (supplementary Tables 1 and 2) were similar to those reported in growing pigs [23], ranging from 31.93 to 34.21 L/(d·BW^0.75^) and 30.99 to 32.42 L/(d·BW^0.75^) for metabolic CO_2_ production and O_2_ consumption, respectively. In addition, daily TH*P* values were similar those reported by Jakobsen et al. [24] who estimated an average THP of 686 kJ/(d·BW^0.75^) for individual lactating sows fed diets containing 188 g/kg CP by indirect calorimetry and double labeled water technique. Cabezón et al. [25] reported a model predicted-value of 745 kJ/(d·BW^0.75^) for parity 3–5 sows and assuming a BW of 250 kg. These findings are in line with results of the current study. Earlier on, Bond et al. [26] measured THP of lactating sows, including their litters at 385 kJ/(d·BW^0.75^) using indirect calorimetry, reflecting lower lactation demand relative to the current study and others. Brown-Brandl et al. [10] and Stinn and Xin [27] reported THP values ranging from 808 to 1418 kJ/(d·BW^0.75^) and from 1188 to 1695 kJ·/(d·BW^0.75^), respectively. In both of these studies, calorimetry was conducted at the facility level, hence the THP values include sows with their litters which are expected to be higher than for individual sows. In the current study, results of daily THP including sows and litters (Supplementary Table 7) were also higher than those of sows alone (Table 7).

The lower MUN concentration for sows fed LCP diets under both TN and HS conditions resulted from less oxidation of excessive dietary AA and urea synthesis [14, 17, 28], implying lower heat associated with LCP diet. In fact, sows fed the LCP diet produced less daily metabolic heat than those fed the HCP diet throughout lactation, in particular on day 18 under HS conditions. The alleviation of body temperature without an increase in RR under HS conditions indicates that body temperature reduction was diet-induced rather than a result of improved thermoregulation. The estimated THP values of lactating sows based on energy balance [15] were 711 and 586 kJ/(d·BW^0.75^) with decreasing dietary CP from 187 to 138 g/kg, respectively. In the present study, THP generated from indirect calorimetry decreased from 649 to 582 kJ/(d·BW^0.75^) under TN conditions, and from 657 to 590 kJ/(d·BW^0.75^) under HS conditions by feeding the same diets. Thus, this study validates the estimated values by Zhang et al. [15]. In growing-finishing pigs, Kerr et al. [14] reported that decreasing dietary protein from 160 to 120 g/kg reduced THP from 690 to 669 kJ/(d·BW^0.75^) under TN conditions, respectively, and from 615 to 569 kJ/(d·BW^0.75^) under HS conditions, respectively. Le Bellego et al. [13] reported a reduction in THP from 1494 to 1393 kJ/(d·BW^0.65^) in response to decreasing dietary CP from 190 to 120 g/kg, respectively. Herein, a reduction of THP in response to decreasing dietary CP were 12.4 and 13.5 kJ/g CP under TN and HS, respectively. Such values for growing-finishing pigs were up to 7.5 and 20.5 kJ/g CP reduction under TN and HS conditions, respectively [13, 14, 29]. In the study by Kerr et al. [14], pigs under HS had a lower feed intake than those under TN because they were not pair-fed. Thus, it is possible that the difference in feed intake contributed to a larger reduction in heat (20.5 kJ/g CP) compared to values reported herein (13.5 kJ/g CP). Under either TN or HS conditions, both average daily feed intake and milk production did not differ between HCP and LCP diets. Therefore the lower THP in sows fed the LCP diet compared to the HCP diet on lactation day 18 was likely attributed to reduced oxidation of excessive dietary AA and urea synthesis [14, 17, 28]. The theoretical heat reduction associated with less AA intake was 1439 kJ/d [28]. This value was estimated using the NE model for the growing-finishing pig, and thus excluded heat associated with mammary metabolism.

The relationship between THP and days in lactation in this study was previously reported by others [10, 12, 27], and followed a similar trend to that of milk production, piglet growth and nutrient demand [22]. Toner et al. [30] described the milk production curve, composed of the colostral, ascending, plateau and descending phases, with duration of the ascending phase varying from day 14 to 28 of lactation, depending on breed, nutrition, parity, and other factors [31, 32]. Hansen et al. [33] reported a mean time to peak lactation of 18.7 days from a meta-analysis study. Increasing THP with progression of lactation, followed by a descending trend, reflects THP associated with lactation demand. The respiratory quotient (RQ = CO_2_ output: O_2_ input) values in this study (supplementary Tables 3 and 6) remained close to 1 throughout lactation, indicating that dietary carbohydrates were serving as primary oxidative substrate [34], and that sows were not in severe negative energy balance. A RQ close to 1 was also previously reported at fed state in growing pigs [10, 23, 35, 36], gestating sows [27, 37] and lactating sows [12, 24, 27].

The lower THP during nighttime compared to daytime, regardless of environmental conditions, was expected and similar to previous findings [10, 27]. This response was likely due to lower feed intake and activity level, as previously described [38], and also due to circadian rhythm differences between daytime and nighttime [10]. Reduced THP between daytime and nighttime corresponded to a 19% and 16% decrease under TN and HS conditions, respectively. Stinn and Xin [27] reported a daytime to nighttime THP reduction of 27% and 6% during late gestation and lactation, respectively, in sows housed at 20 °C.

In addition to less AA oxidation, the lower thermic effect of feeding supplemental AA versus intact protein may possibly contribute to lower THP in LCP diet [16]. To our knowledge, heat increment measurement in lactating sows has not previously been reported. In this study, THP measured at different time points during the day was not affected by the feeding schedule (07:00, 13:00 and 19:00), which was likely attributed to short duration of time between feedings. The longest time between air sampling measurements was 12 h, between the last evening feeding at 19:00 and the morning feeding at 07:00. In growing-finishing pigs, THP was reported to differ between pre- and post-feeding under feed restriction exceeding 30 h [35, 36]. In these studies, the RQ decreased to 0.8, suggesting oxidation of body protein and adipose tissues [34] and pointing to a fasted state [39]. Note that in this study, the RQ before the morning feeding was approximately 1 (see supplementary Table 6), suggesting the major substrate for oxidation was glucose with blood glucose level maintained by hepatic glycogen availability [40]. Therefore, 12 h fasting overnight was not sufficient to elicit a fasting state, despite the high metabolic demands of lactation.

Animals under high ambient temperature reduce their metabolic heat production and improve heat losses by latent and sensible pathways [5]. Thus, reduced feed intake, milk production or growth rate have been considered as adaptive mechanisms to high ambient temperature through mitigation of metabolic heat [5]. It was traditionally recognized that maintenance cost increases under HS in ruminants [41], rodents [42] and swine [43], as a result of greater energy associated with heat dissipation, such as panting. Conversely, using a comparative slaughter technique, Johnson et al. [44] estimated that pigs exposed to HS require 2.5 MJ/d less ME for maintenance than pigs raised under TN conditions. Yunianto et al. [45] also reported lower heat production and reduced plasma triiodothyronine (T3), thyroxine (T4) in broiler chickens under HS than TN. Lower THP under HS was also found in growing pigs [14, 46, 47]. Heat reduction under HS may be related to lower visceral mass [48] or feed intake [46]. In lactating sows, in addition to milk nutrient synthesis, the main contributor to THP is heat increment of feeding [16, 49]. Herein, it was initially planned to pair-feed TN sows to preceding HS sows in order to compare THP between TN and HS. However, feed intake between diets varied within either TN or HS environment, thus MEI was included as a covariable under HS to adjust THP. The MEI under TN was fixed due to pair feeding, and was not an independent and random variable, thus the covariable MEI was not included under TN. Thus, the THP under TN and HS was not compared since THP was analyzed with a different model (i.e., TN without covariable MEI and HS with covariable MEI).

Sows fed the LCP diet lost more BW than those fed the HCP diet under HS conditions, which may be attributed to greater body protein mobilization. Increased partitioning of AA towards the mammary gland at the expense of maternal body reserves in sows fed an LCP diet has been suggested by Huber et al. [50]. Long term exposure to HS environment may further aggravate skeletal muscle catabolism [51–53]. The loss of BW and protein reserve is of potential concern for subsequent reproductive cycle [54] and therefore additional research is needed to evaluate the feasibility of feeding an LCP diet over several parities. Similar findings have been reported under TN conditions [17, 50, 55]. On the other hand, others reported that sow BW loss did not differ between HCP and LCP diets under HS conditions [56, 57]. Of note, sows fed HCP diets lost less BW under HS compared to the pair fed TN (PFTN) counterparts in peak lactation, with similar results observed in gilts fed 175 g/kg CP [52]. Thus, PFTN animals may be under greater physiological stress compared to their HS counterparts due to nutrient restriction [52]. Although not observed in this study due to lack of power, the lower THP under HS conditions [5] may lead to more dietary energy partitioning towards maintaining maternal BW compared to PFTN. In the latter, the greater BW loss of PFTN counterparts fed 175 g/kg CP was also due to body protein loss. Conversely, when fed the LCP diet, sows in the current study tended to lose more BW and body protein under HS compared to their PFTN counterparts, suggesting an interaction between diet and environment. It is possible that the LCP diet was limiting in certain AA under HS condition. For instance, AA oxidation increases due to greater maintenance cost under HS conditions [43]. In addition, lactating sows exposed to HS conditions were reported to have reduced milk concentration of arginine, valine and proline [3]. These observations [3, 43] suggest that HS may increase oxidation of certain AA and as a result, may lead to AA imbalance.

## Conclusion

Feeding reduced CP diets with a NIAA profile alleviated the increased body temperature of sows under HS environment which was accompanied by a reduction in respiration rate. Feeding LCP diets reduced daily THP by 10.3% over the lactation period, and this reduction was associated with the THP response on day 18 of lactation. Sows fed LCP diet had 73% average reduction in MUN and maintained similar feed intake and lactation performance compared to sows fed HCP, suggesting that reduction of THP in sows fed LCP was attributed to less oxidation of excessive dietary AA and reduced urea synthesis. Total heat production is associated with days in lactation, in particular under HS conditions with THP appearing to peak between days 14 and 18.

Results suggest that sows under HS environment and fed reduced dietary CP with a NIAA balance demonstrated less physiological stress to heat. The reduction of THP also implies an increased dietary energy utilization efficiency for lactation during the later stage of lactation. Zhang et al. [15] also indicated the efficiency of energy utilization based on energy balance data and estimated heat production was greater in the peak stage of lactation in sows fed a NIAA profile diet. These results shed additional light on the potential benefits of feeding low protein diets on a larger scale, including maximizing production efficiency, improving welfare of lactating sows under global warming and potentially mitigating the carbon footprint. Amino acid requirements of lactating sows exposed to HS will need to be re-evaluated in order to formulate diets with NIAA profile that maintain maternal body protein retention in order to implement such nutritional strategy over multiple parities.

## Supplementary information

**Additional file 1:Table S1**. Metabolic oxygen (O_2_) consumption [L/(d·BW^0.75^)] of lactating sows fed high crude protein (HCP) and low crude protein (LCP) diet and exposed to thermal neutral and heat stress conditions. **Table S2**. Metabolic carbon dioxide (CO_2_) production [L/(d·BW^0.75^)]of lactating sows fed high crude protein (HCP) and low crude protein (LCP) diet and exposed to thermal neutral and heat stress conditions. **Table S3**. Respiratory quotient (RQ) of lactating sows fed high crude protein (HCP) and low crude protein (LCP) diet and exposed to thermal neutral and heat stress conditions. **Table S4**. Metabolic oxygen (O_2_) consumption [L/(d·BW^0.75^)]during daytime of lactating sows fed high crude protein (HCP) and low crude protein (LCP) diet and exposed to thermal neutral and heat stress conditions. **Table S5**. Metabolic carbon dioxide (CO_2_) production [L/(d·BW^0.75^)] during daytime of lactating sows fed high crude protein (HCP) and low crude protein (LCP) diet and exposed to thermal neutral and heat stress conditions. **Table S6**. Respiratory quotient (RQ) during daytime of lactating sows fed high crude protein (HCP) and low crude protein (LCP) diet and exposed to thermal neutral and heat stress conditions. **Table S7**. Metabolic total heat production [kJ/(d·BW^0.75^)] of lactating sows with litters fed high crude protein (HCP) and low crude protein (LCP) diet and exposed to thermal neutral and heat stress conditions. **Table S8**. Metabolic oxygen (O_2_) consumption [L/(d·BW^0.75^)] of lactating sows with litters fed high crude protein (HCP) and low crude protein (LCP) diet and exposed to thermal neutral and heat stress conditions. **Table S9**. Metabolic carbon dioxide (CO_2_) production [L/(d·BW^0.75^)] of lactating sows with litters fed high crude protein (HCP) and low crude protein (LCP) diet and exposed to thermal neutral and heat stress conditions. **Table S10**. Respiratory quotient (RQ) of lactating sows with litters fed high crude protein (HCP) and low crude protein (LCP) diet and exposed to thermal neutral and heat stress conditions. **Table S11**. Metabolic total heat production [kJ/(d·BW^0.75^)] during daytime of lactating sows with litters fed high crude protein (HCP) and low crude protein (LCP) diet and exposed to thermal neutral and heat stress conditions. **Table S12**. Metabolic oxygen (O_2_) consumption [L/(d·BW^0.75^)] during daytime of lactating sows with litters fed high crude protein (HCP) and low crude protein (LCP) diet and exposed to thermal neutral and heat stress conditions. **Table S13**. Metabolic carbon dioxide (CO_2_) production [L/(d·BW^0.75^)] during daytime of lactating sows with litters fed high crude protein (HCP) and low crude protein (LCP) diet and exposed to thermal neutral and heat stress conditions. **Table S14**. Respiratory quotient (RQ) during daytime of lactating sows with litters fed high crude protein (HCP) and low crude protein (LCP) diet and exposed to thermal neutral and heat stress conditions. **Table S15**. Metabolic carbon dioxide (CO_2_) production, oxygen (O_2_) consumption, total heat production (THP) and respiratory quotient (RQ) of piglets from sows fed high crude protein (HCP) and low crude protein (LCP) diet and exposed to thermal neutral and heat stress conditions.

## Data Availability

The datasets used and/or analyzed during the current study are available from the corresponding author on request.

## Abbreviations

AA: Amino acid
BP: Body protein
BW: Body weight
CP: Crude protein
DM: Dry matter
HCP: High crude protein
HS: Heat stress
IDAA: Indispensable amino acid
LCP: Low crude protein
LW: Litter weight
MEI: Metabolizable energy intake
MUN: Milk urea nitrogen
NE: Net energy
NIAA: Near ideal amino acid profile
RQ: Respiratory quotient
RR: Respiration rate
SID: Standardized ileal digestibility coefficient
THP: Total heat production
TN: Thermal neutral
