# Supplementary material for: Effect of dietary near ideal amino acid profile on heat production of lactating sows exposed to thermal neutral and heat stress conditions
Source: J Anim Sci Biotechnol. 2020 Jul 9;11:75. doi: 10.1186/s40104-020-00483-w (PMC7346526; doi:10.1186/s40104-020-00483-w)
Supplement: Supplementary file 1 — Additional file 1:Table S1. Metabolic oxygen (O2) consumption [L/(d·BW0.75)] of lactating sows fed high crude protein (HCP) and low crude protein (LCP) diet and exposed to thermal neutral and heat stress conditions. Table S2. Metabolic carbon dioxide (CO2) production [L/(d·BW0.75)]of lactating sows fed high crude protein (HCP) and low crude protein (LCP) diet and exposed to thermal neutral and heat stress conditions. Table S3. Respiratory quotient (RQ) of lactating sows fed high crude protein (HCP) and low crude protein (LCP) diet and exposed to thermal neutral and heat stress conditions. Table S4. Metabolic oxygen (O2) consumption [L/(d·BW0.75)]during daytime of lactating sows fed high crude protein (HCP) and low crude protein (LCP) diet and exposed to thermal neutral and heat stress conditions. Table S5. Metabolic carbon dioxide (CO2) production [L/(d·BW0.75)] during daytime of lactating sows fed high crude protein (HCP) and low crude protein (LCP) diet and exposed to thermal neutral and heat stress conditions. Table S6. Respiratory quotient (RQ) during daytime of lactating sows fed high crude protein (HCP) and low crude protein (LCP) diet and exposed to thermal neutral and heat stress conditions. Table S7. Metabolic total heat production [kJ/(d·BW0.75)] of lactating sows with litters fed high crude protein (HCP) and low crude protein (LCP) diet and exposed to thermal neutral and heat stress conditions. Table S8. Metabolic oxygen (O2) consumption [L/(d·BW0.75)] of lactating sows with litters fed high crude protein (HCP) and low crude protein (LCP) diet and exposed to thermal neutral and heat stress conditions. Table S9. Metabolic carbon dioxide (CO2) production [L/(d·BW0.75)] of lactating sows with litters fed high crude protein (HCP) and low crude protein (LCP) diet and exposed to thermal neutral and heat stress conditions. Table S10. Respiratory quotient (RQ) of lactating sows with litters fed high crude protein (HCP) and low crude protein (LCP) diet and exposed [file 40104_2020_483_MOESM1_ESM.docx]

Table S1. Metabolic oxygen (O_2_) consumption [L/(d·BW^0.75^)] of lactating sows fed high crude protein (HCP) and low crude protein (LCP) diet and exposed to thermal neutral and heat stress conditions^1^

| Item | Thermal neutral | | | |  | Heat stress | | | |
| --- | --- | --- | --- | --- | --- | --- | --- | --- | --- |
|  | HCP | LCP | SEM^2^ | *P*-value |  | HCP | LCP | SEM^2^ | *P*-value |
| Nighttime^4^ | | | | |  |  |  |  |  |
| Day 4 | 25.49 | 20.02 | 2.77 | 0.171 |  | 27.89 | 25.09 | 2.05 | 0.254 |
| Day 8 | 25.49 | 23.33 | 2.77 | 0.585 |  | 27.85 | 24.46 | 1.74 | 0.134 |
| Day 14 | 29.81 | 25.49 | 2.77 | 0.277 |  | 28.44 | 26.70 | 1.77 | 0.444 |
| Day 18 | 27.07 | 21.43 | 2.77 | 0.178 |  | 25.70 | 20.27 | 1.74 | 0.017 |
| Daytime | | | | |  |  |  |  |  |
| Day 4 | 29.64 | 29.38 | 2.33 | 0.910 |  | 31.73 | 31.56 | 1.49 | 0.932 |
| Day 8 | 34.56 | 32.40 | 2.33 | 0.397 |  | 32.88 | 31.45 | 1.33 | 0.459 |
| Day 14 | 37.32 | 31.80 | 2.33 | 0.036 |  | 32.73 | 31.26 | 1.36 | 0.436 |
| Day 18 | 34.30 | 31.19 | 2.33 | 0.254 |  | 33.31 | 25.62 | 1.34 | <0.001 |
| 24 h | | | | |  |  |  |  |  |
| Day 4 | 27.48 | 24.62 | 2.25 | 0.321 |  | 30.12 | 28.26 | 1.46 | 0.319 |
| Day 8 | 29.98 | 27.91 | 2.25 | 0.485 |  | 30.05 | 27.93 | 1.31 | 0.248 |
| Day 14 | 33.52 | 28.68 | 2.25 | 0.095 |  | 30.65 | 29.28 | 1.34 | 0.440 |
| Day 18 | 30.84 | 26.09 | 2.25 | 0.122 |  | 29.49 | 22.75 | 1.32 | 0.001 |

^1^Data are least squares means.

^2^Maximum value of the standard error of the means.

Table S2. Metabolic carbon dioxide (CO_2_) production [L/(d·BW^0.75^)]of lactating sows fed high crude protein (HCP) and low crude protein (LCP) diet and exposed to thermal neutral and heat stress conditions^1^

|  | Thermal neutral | | | |  | Heat stress | | | |
| --- | --- | --- | --- | --- | --- | --- | --- | --- | --- |
|  | HCP | LCP | SEM^2^ | *P*-value |  | HCP | LCP | SEM^2^ | *P*-value |
| Nighttime^4^ | | | | |  |  |  |  |  |
| Day 4 | 29.52 | 28.23 | 2.98 | 0.748 |  | 28.70 | 21.68 | 3.63 | 0.046 |
| Day 8 | 31.25 | 30.82 | 2.98 | 0.915 |  | 28.62 | 28.97 | 3.28 | 0.909 |
| Day 14 | 33.84 | 32.55 | 2.98 | 0.748 |  | 32.81 | 34.69 | 3.32 | 0.556 |
| Day 18 | 34.85 | 29.67 | 2.98 | 0.226 |  | 30.96 | 26.17 | 3.29 | 0.124 |
| Daytime | | | | |  |  |  |  |  |
| Day 4 | 29.66 | 28.94 | 2.90 | 0.805 |  | 32.61 | 32.75 | 1.72 | 0.948 |
| Day 8 | 33.98 | 31.97 | 2.90 | 0.490 |  | 31.94 | 30.06 | 1.54 | 0.398 |
| Day 14 | 35.57 | 34.13 | 2.90 | 0.621 |  | 32.97 | 32.25 | 1.57 | 0.743 |
| Day 18 | 33.84 | 31.84 | 2.90 | 0.513 |  | 34.08 | 26.02 | 1.54 | <0.001 |
| 24 h | | | | |  |  |  |  |  |
| Day 4 | 29.38 | 28.51 | 2.69 | 0.783 |  | 31.02 | 27.50 | 1.92 | 0.113 |
| Day 8 | 32.69 | 31.25 | 2.69 | 0.647 |  | 30.01 | 29.57 | 1.77 | 0.836 |
| Day 14 | 34.71 | 33.26 | 2.69 | 0.647 |  | 32.89 | 33.00 | 1.80 | 0.960 |
| Day 18 | 34.27 | 31.03 | 2.69 | 0.329 |  | 32.60 | 26.20 | 1.78 | 0.004 |

^1^Data are least squares means.

^2^Maximum value of the standard error of the means.

Table S3. Respiratory quotient (RQ) of lactating sows fed high crude protein (HCP) and low crude protein (LCP) diet and exposed to thermal neutral and heat stress conditions^1^

| Item | Thermal neutral | | | |  | Heat stress | | | |
| --- | --- | --- | --- | --- | --- | --- | --- | --- | --- |
|  | HCP | LCP | SEM^2^ | *P*-value |  | HCP | LCP | SEM^2^ | *P*-value |
| Nighttime | | | | |  |  |  |  |  |
| Day 4 | 1.24 | 1.45 | 0.12 | 0.229 |  | 1.00 | 0.86 | 0.13 | 0.287 |
| Day 8 | 1.29 | 1.36 | 0.12 | 0.709 |  | 1.04 | 1.22 | 0.11 | 0.156 |
| Day 14 | 1.15 | 1.32 | 0.12 | 0.306 |  | 1.16 | 1.31 | 0.11 | 0.254 |
| Day 18 | 1.30 | 1.41 | 0.12 | 0.525 |  | 1.20 | 1.34 | 0.11 | 0.247 |
| Daytime | | | | |  |  |  |  |  |
| Day 4 | 1.01 | 1.01 | 0.04 | 0.940 |  | 1.06 | 1.05 | 0.03 | 0.700 |
| Day 8 | 0.98 | 1.02 | 0.04 | 0.443 |  | 0.97 | 0.96 | 0.03 | 0.688 |
| Day 14 | 0.96 | 1.08 | 0.04 | 0.011 |  | 1.01 | 1.03 | 0.03 | 0.438 |
| Day 18 | 1.00 | 1.03 | 0.04 | 0.673 |  | 1.03 | 1.03 | 0.03 | 0.921 |
| 24 h | | | | |  |  |  |  |  |
| Day 4 | 1.12 | 1.23 | 0.06 | 0.218 |  | 1.05 | 0.96 | 0.05 | 0.185 |
| Day 8 | 1.14 | 1.19 | 0.06 | 0.553 |  | 1.01 | 1.09 | 0.05 | 0.200 |
| Day 14 | 1.05 | 1.20 | 0.06 | 0.081 |  | 1.08 | 1.14 | 0.05 | 0.365 |
| Day 18 | 1.15 | 1.22 | 0.06 | 0.449 |  | 1.11 | 1.19 | 0.05 | 0.259 |

^1^Data are least squares means.

^2^Maximum value of the standard error of the means.

Table S4. Metabolic oxygen (O_2_) consumption [L/(d·BW^0.75^)]during daytime of lactating sows fed high crude protein (HCP) and low crude protein (LCP) diet and exposed to thermal neutral and heat stress conditions^1^

| Item | Thermal neutral | | | |  | Heat stress | | | |
| --- | --- | --- | --- | --- | --- | --- | --- | --- | --- |
|  | HCP | LCP | SEM^2^ | *P*-value |  | HCP | LCP | SEM^2^ | *P*-value |
| Day 4 |  |  |  |  |  |  |  |  |  |
| 07:00^3^ | 29.81 | 30.80 | 2.53 | 0.747 |  | 32.62 | 30.81 | 3.02 | 0.655 |
| 08:00 | 29.66 | 31.31 | 2.53 | 0.595 |  | 31.18 | 29.93 | 3.02 | 0.757 |
| 09:00 | 27.22 | 29.63 | 2.53 | 0.437 |  | 30.61 | 33.77 | 3.02 | 0.438 |
| 10:00 | 28.66 | 29.56 | 2.53 | 0.771 |  | 31.03 | 31.08 | 3.02 | 0.991 |
| 11:00 | 30.09 | 28.49 | 2.53 | 0.603 |  | 29.96 | 29.64 | 3.02 | 0.937 |
| 13:00 | 31.82 | 35.35 | 2.53 | 0.258 |  | 30.74 | 26.66 | 3.02 | 0.316 |
| 15:00 | 31.10 | 28.93 | 2.53 | 0.483 |  | 32.45 | 29.40 | 3.41 | 0.484 |
| 19:00 | 28.51 | 29.44 | 2.70 | 0.774 |  | 21.77 | 28.57 | 3.41 | 0.123 |
| Day 8 |  |  |  |  |  |  |  |  |  |
| 07:00^3^ | 32.40 | 27.93 | 3.03 | 0.262 |  | 33.28 | 26.69 | 2.24 | 0.045 |
| 08:00 | 35.57 | 34.41 | 3.03 | 0.771 |  | 35.44 | 32.60 | 2.24 | 0.378 |
| 09:00 | 31.97 | 28.23 | 3.03 | 0.346 |  | 34.15 | 31.01 | 2.24 | 0.332 |
| 10:00 | 34.85 | 31.82 | 3.03 | 0.446 |  | 31.70 | 28.85 | 2.24 | 0.378 |
| 11:00 | 34.13 | 33.26 | 3.03 | 0.827 |  | 32.85 | 31.44 | 2.24 | 0.662 |
| 13:00 | 35.57 | 34.41 | 3.03 | 0.771 |  | 33.86 | 28.85 | 2.24 | 0.124 |
| 15:00 | 37.58 | 36.14 | 3.03 | 0.717 |  | 37.03 | 31.59 | 2.24 | 0.096 |
| 19:00 | 34.27 | 32.55 | 3.03 | 0.663 |  | 31.55 | 34.61 | 2.24 | 0.344 |
| Day 14 |  |  |  |  |  |  |  |  |  |
| 07:00^3^ | 39.60 | 30.82 | 2.73 | 0.015 |  | 36.63 | 32.88 | 2.56 | 0.298 |
| 08:00 | 35.71 | 32.98 | 2.73 | 0.437 |  | 35.92 | 36.90 | 2.56 | 0.783 |
| 09:00 | 34.99 | 30.53 | 2.73 | 0.207 |  | 34.33 | 30.85 | 2.56 | 0.336 |
| 10:00 | 35.71 | 29.95 | 2.73 | 0.105 |  | 35.34 | 28.69 | 2.56 | 0.069 |
| 11:00 | 34.99 | 32.40 | 2.73 | 0.461 |  | 35.92 | 32.73 | 2.56 | 0.378 |
| 13:00 | 38.02 | 31.54 | 2.73 | 0.069 |  | 27.85 | 29.46 | 2.56 | 0.665 |
| 15:00 | 40.61 | 36.87 | 2.73 | 0.289 |  | 34.33 | 33.45 | 2.56 | 0.806 |
| 19:00 | 37.58 | 30.09 | 2.73 | 0.037 |  | 32.89 | 31.29 | 2.56 | 0.657 |
| Day 18 |  |  |  |  |  |  |  |  |  |
| 07:00^3^ | 36.29 | 32.25 | 3.63 | 0.390 |  | 35.42 | 29.51 | 2.61 | 0.115 |
| 08:00 | 38.59 | 32.73 | 3.63 | 0.213 |  | 36.14 | 25.92 | 2.61 | 0.008 |
| 09:00 | 31.25 | 28.79 | 3.63 | 0.600 |  | 30.66 | 25.34 | 2.61 | 0.154 |
| 10:00 | 29.66 | 26.06 | 3.63 | 0.443 |  | 30.38 | 20.87 | 2.61 | 0.013 |
| 11:00 | 31.54 | 30.52 | 3.63 | 0.828 |  | 33.98 | 23.61 | 2.61 | 0.007 |
| 13:00 | 34.56 | 31.20 | 3.63 | 0.475 |  | 40.38 | 26.28 | 2.84 | 0.001 |
| 15:00 | 38.45 | 34.64 | 3.63 | 0.417 |  | 36.94 | 24.88 | 2.85 | 0.009 |
| 19:00 | 33.98 | 29.93 | 3.63 | 0.388 |  | 31.95 | 27.648 | 2.86 | 0.310 |

^1^Data are least squares means.

^2^Maximum value of the standard error of the means.

^3^Prior to morning feeding at 07:00.

Table S5. Metabolic carbon dioxide (CO_2_) production [L/(d·BW^0.75^)] during daytime of lactating sows fed high crude protein (HCP) and low crude protein (LCP) diet and exposed to thermal neutral and heat stress conditions^1^

| Item | Thermal neutral | | | |  | Heat stress | | | |
| --- | --- | --- | --- | --- | --- | --- | --- | --- | --- |
|  | HCP | LCP | SEM^2^ | *P*-value |  | HCP | LCP | SEM^2^ | *P*-value |
| Day 4 |  |  |  |  |  |  |  |  |  |
| 07:00^3^ | 28.66 | 27.10 | 2.75 | 0.626 |  | 32.92 | 30.13 | 2.34 | 0.379 |
| 08:00 | 30.82 | 29.33 | 2.75 | 0.643 |  | 33.63 | 35.45 | 2.34 | 0.565 |
| 09:00 | 27.36 | 29.50 | 2.75 | 0.506 |  | 33.09 | 36.18 | 2.34 | 0.333 |
| 10:00 | 28.94 | 27.79 | 2.75 | 0.718 |  | 30.92 | 29.83 | 2.34 | 0.731 |
| 11:00 | 30.39 | 29.18 | 2.75 | 0.706 |  | 32.26 | 31.42 | 2.53 | 0.799 |
| 13:00 | 32.40 | 30.87 | 2.75 | 0.633 |  | 32.22 | 32.14 | 2.34 | 0.981 |
| 15:00 | 30.53 | 30.40 | 2.75 | 0.968 |  | 33.44 | 29.98 | 2.55 | 0.299 |
| 19:00 | 28.51 | 29.78 | 2.86 | 0.699 |  | 31.80 | 27.82 | 2.57 | 0.235 |
| Day 8 |  |  |  |  |  |  |  |  |  |
| 07:00^3^ | 29.38 | 30.39 | 2.94 | 0.780 |  | 31.20 | 27.06 | 2.23 | 0.198 |
| 08:00 | 34.71 | 33.12 | 2.94 | 0.661 |  | 35.09 | 30.08 | 2.23 | 0.121 |
| 09:00 | 32.69 | 31.10 | 2.94 | 0.661 |  | 32.63 | 33.26 | 2.23 | 0.847 |
| 10:00 | 33.98 | 30.82 | 2.94 | 0.382 |  | 32.20 | 28.94 | 2.23 | 0.308 |
| 11:00 | 34.13 | 31.82 | 2.94 | 0.524 |  | 32.50 | 30.80 | 2.23 | 0.597 |
| 13:00 | 36.14 | 33.41 | 2.94 | 0.450 |  | 31.92 | 26.48 | 2.23 | 0.093 |
| 15:00 | 36.57 | 32.40 | 2.94 | 0.251 |  | 35.66 | 29.07 | 2.23 | 0.043 |
| 19:00 | 34.99 | 30.96 | 2.94 | 0.267 |  | 29.47 | 30.52 | 2.23 | 0.743 |
| Day 14 |  |  |  |  |  |  |  |  |  |
| 07:00^3^ | 35.42 | 31.68 | 2.93 | 0.302 |  | 32.58 | 32.70 | 2.26 | 0.969 |
| 08:00 | 35.14 | 33.70 | 2.93 | 0.689 |  | 36.61 | 36.59 | 2.26 | 0.995 |
| 09:00 | 33.98 | 33.41 | 2.93 | 0.873 |  | 36.32 | 32.56 | 2.26 | 0.239 |
| 10:00 | 34.27 | 33.55 | 2.93 | 0.842 |  | 34.59 | 30.26 | 2.26 | 0.175 |
| 11:00 | 34.27 | 34.27 | 2.93 | 1.000 |  | 35.46 | 34.15 | 2.26 | 0.678 |
| 13:00 | 35.42 | 31.54 | 2.93 | 0.284 |  | 28.40 | 29.53 | 2.26 | 0.722 |
| 15:00 | 40.03 | 39.60 | 2.93 | 0.904 |  | 34.74 | 33.13 | 2.26 | 0.613 |
| 19:00 | 36.57 | 35.28 | 2.93 | 0.719 |  | 33.73 | 32.56 | 2.26 | 0.712 |
| Day 18 |  |  |  |  |  |  |  |  |  |
| 07:00^3^ | 31.54 | 30.27 | 3.36 | 0.761 |  | 33.85 | 28.43 | 2.31 | 0.102 |
| 08:00 | 35.71 | 33.85 | 3.36 | 0.653 |  | 36.59 | 27.13 | 2.31 | 0.005 |
| 09:00 | 32.83 | 31.19 | 3.36 | 0.691 |  | 32.56 | 23.53 | 2.31 | 0.008 |
| 10:00 | 31.68 | 27.57 | 3.36 | 0.323 |  | 31.69 | 23.24 | 2.31 | 0.012 |
| 11:00 | 33.26 | 31.58 | 3.36 | 0.683 |  | 35.01 | 23.82 | 2.31 | 0.001 |
| 13:00 | 34.56 | 32.44 | 3.36 | 0.610 |  | 38.02 | 27.03 | 2.50 | 0.004 |
| 15:00 | 36.43 | 34.69 | 3.36 | 0.674 |  | 37.23 | 26.87 | 2.52 | 0.007 |
| 19:00 | 34.85 | 29.19 | 3.36 | 0.175 |  | 33.69 | 28.50 | 2.52 | 0.167 |

^1^Data are least squares means.

^2^Maximum value of the standard error of the means.

^3^Prior to morning feeding at 07:00.

Table S6. Respiratory quotient (RQ) during daytime of lactating sows fed high crude protein (HCP) and low crude protein (LCP) diet and exposed to thermal neutral and heat stress conditions^1^

| Item | Thermal neutral | | | |  | Heat stress | | | |
| --- | --- | --- | --- | --- | --- | --- | --- | --- | --- |
|  | HCP | LCP | SEM^2^ | *P*-value |  | HCP | LCP | SEM^2^ | *P*-value |
| Day 4 |  |  |  |  |  |  |  |  |  |
| 07:00^3^ | 0.97 | 0.89 | 0.05 | 0.244 |  | 1.07 | 1.00 | 0.05 | 0.284 |
| 08:00 | 1.04 | 0.93 | 0.05 | 0.120 |  | 1.08 | 1.15 | 0.05 | 0.277 |
| 09:00 | 1.01 | 1.01 | 0.05 | 0.999 |  | 1.07 | 1.10 | 0.05 | 0.553 |
| 10:00 | 1.02 | 0.95 | 0.05 | 0.331 |  | 1.00 | 1.01 | 0.05 | 0.945 |
| 11:00 | 1.01 | 1.03 | 0.05 | 0.804 |  | 1.05 | 1.06 | 0.05 | 0.846 |
| 13:00 | 1.02 | 0.88 | 0.05 | 0.046 |  | 1.04 | 1.11 | 0.05 | 0.230 |
| 15:00 | 0.99 | 1.05 | 0.05 | 0.418 |  | 1.06 | 1.05 | 0.06 | 0.896 |
| 19:00 | 0.99 | 1.00 | 0.06 | 0.962 |  | 1.02 | 1.00 | 0.07 | 0.732 |
| Day 8 |  |  |  |  |  |  |  |  |  |
| 07:00^3^ | 0.89 | 1.21 | 0.09 | 0.014 |  | 0.94 | 1.02 | 0.04 | 0.131 |
| 08:00 | 0.97 | 0.95 | 0.09 | 0.868 |  | 1.00 | 0.92 | 0.04 | 0.134 |
| 09:00 | 1.01 | 1.25 | 0.09 | 0.064 |  | 0.96 | 1.06 | 0.04 | 0.073 |
| 10:00 | 0.97 | 0.96 | 0.09 | 0.925 |  | 1.01 | 1.00 | 0.04 | 0.759 |
| 11:00 | 1.00 | 0.95 | 0.09 | 0.661 |  | 0.98 | 0.97 | 0.04 | 0.787 |
| 13:00 | 1.02 | 0.97 | 0.09 | 0.711 |  | 0.95 | 0.91 | 0.04 | 0.504 |
| 15:00 | 0.97 | 0.90 | 0.09 | 0.593 |  | 0.96 | 0.92 | 0.04 | 0.524 |
| 19:00 | 1.02 | 0.95 | 0.09 | 0.588 |  | 0.95 | 0.91 | 0.04 | 0.480 |
| Day 14 |  |  |  |  |  |  |  |  |  |
| 07:00^3^ | 0.90 | 1.04 | 0.04 | 0.018 |  | 0.93 | 1.01 | 0.03 | 0.114 |
| 08:00 | 0.98 | 1.04 | 0.04 | 0.287 |  | 1.02 | 1.00 | 0.03 | 0.675 |
| 09:00 | 0.97 | 1.10 | 0.04 | 0.036 |  | 1.05 | 1.06 | 0.03 | 0.857 |
| 10:00 | 0.95 | 1.13 | 0.04 | 0.004 |  | 0.99 | 1.06 | 0.03 | 0.128 |
| 11:00 | 0.98 | 1.07 | 0.04 | 0.115 |  | 0.98 | 1.06 | 0.03 | 0.119 |
| 13:00 | 0.94 | 1.03 | 0.04 | 0.101 |  | 1.02 | 1.01 | 0.03 | 0.821 |
| 15:00 | 0.98 | 1.09 | 0.04 | 0.066 |  | 1.01 | 1.00 | 0.03 | 0.764 |
| 19:00 | 0.98 | 1.18 | 0.04 | 0.001 |  | 1.02 | 1.06 | 0.03 | 0.486 |
| Day 18 |  |  |  |  |  |  |  |  |  |
| 07:00^3^ | 0.97 | 0.94 | 0.06 | 0.735 |  | 0.97 | 0.96 | 0.04 | 0.853 |
| 08:00 | 0.92 | 1.05 | 0.06 | 0.109 |  | 1.02 | 1.07 | 0.04 | 0.468 |
| 09:00 | 1.05 | 1.06 | 0.06 | 0.843 |  | 1.07 | 0.96 | 0.04 | 0.088 |
| 10:00 | 1.06 | 1.04 | 0.06 | 0.796 |  | 1.06 | 1.12 | 0.04 | 0.317 |
| 11:00 | 1.05 | 1.03 | 0.06 | 0.836 |  | 1.04 | 1.02 | 0.04 | 0.690 |
| 13:00 | 0.99 | 1.07 | 0.06 | 0.371 |  | 0.95 | 1.02 | 0.05 | 0.322 |
| 15:00 | 0.95 | 0.99 | 0.06 | 0.670 |  | 1.00 | 1.12 | 0.05 | 0.131 |
| 19:00 | 1.04 | 0.96 | 0.06 | 0.300 |  | 1.06 | 1.03 | 0.05 | 0.658 |

^1^Data are least squares means.

^2^Maximum value of the standard error of the means.

^3^Prior to morning feeding at 07:00.

Table S7. Metabolic total heat production [kJ/(d·BW^0.75^)] of lactating sows with litters fed high crude protein (HCP) and low crude protein (LCP) diet and exposed to thermal neutral and heat stress conditions^1^

| Item | Thermal neutral | | | |  | Heat stress | | | |
| --- | --- | --- | --- | --- | --- | --- | --- | --- | --- |
|  | HCP | LCP | SEM^2^ | *P*-value |  | HCP | LCP | SEM^2^ | *P*-value |
| Nighttime | | | | |  |  |  |  |  |
| Day 4 | 672.8 | 577.4 | 52.3 | 0.176 |  | 695.0 | 584.5 | 46.0 | 0.033 |
| Day 8 | 715.9 | 677.8 | 52.3 | 0.583 |  | 725.9 | 659.8 | 40.2 | 0.152 |
| Day 14 | 832.6 | 774.0 | 52.3 | 0.403 |  | 816.3 | 769.0 | 41.0 | 0.322 |
| Day 18 | 845.6 | 733.9 | 52.3 | 0.133 |  | 788.3 | 730.1 | 41.0 | 0.201 |
| Daytime | | | | |  |  |  |  |  |
| Day 4 | 730.9 | 723.0 | 51.0 | 0.859 |  | 776.1 | 733.9 | 31.0 | 0.314 |
| Day 8 | 857.7 | 810.4 | 51.0 | 0.334 |  | 823.4 | 767.3 | 28.0 | 0.167 |
| Day 14 | 939.7 | 867.8 | 51.0 | 0.144 |  | 877.8 | 829.3 | 28.0 | 0.227 |
| Day 18 | 936.8 | 872.8 | 53.1 | 0.216 |  | 904.6 | 804.2 | 28.0 | 0.015 |
| 24 h | | | | |  |  |  |  |  |
| Day 4 | 702.1 | 649.8 | 48.1 | 0.343 |  | 743.9 | 657.7 | 32.2 | 0.036 |
| Day 8 | 787.4 | 743.9 | 48.1 | 0.435 |  | 774.0 | 712.1 | 29.3 | 0.117 |
| Day 14 | 885.8 | 820.5 | 48.1 | 0.236 |  | 846.4 | 801.2 | 30.1 | 0.239 |
| Day 18 | 891.6 | 804.2 | 51.0 | 0.134 |  | 846.4 | 766.1 | 29.3 | 0.044 |

^1^Data are least squares means.

^2^Maximum value of the standard error of the means.

Table S8. Metabolic oxygen (O_2_) consumption [L/(d·BW^0.75^)] of lactating sows with litters fed high crude protein (HCP) and low crude protein (LCP) diet and exposed to thermal neutral and heat stress conditions^1^

| Item | Thermal neutral | | | |  | Heat stress | | | |
| --- | --- | --- | --- | --- | --- | --- | --- | --- | --- |
|  | HCP | LCP | SEM^2^ | *P*-value |  | HCP | LCP | SEM^2^ | *P*-value |
| Nighttime^4^ | | | | |  |  |  |  |  |
| Day 4 | 31.10 | 25.77 | 2.58 | 0.140 |  | 32.99 | 28.66 | 1.92 | 0.092 |
| Day 8 | 32.98 | 30.39 | 2.58 | 0.468 |  | 34.12 | 30.23 | 1.82 | 0.098 |
| Day 14 | 38.73 | 35.71 | 2.58 | 0.397 |  | 38.18 | 35.43 | 1.96 | 0.245 |
| Day 18 | 39.03 | 33.60 | 2.81 | 0.152 |  | 36.61 | 33.96 | 1.80 | 0.242 |
|  | | | | |  |  |  |  |  |
| Daytime | | | | |  |  |  |  |  |
| Day 4 | 34.85 | 34.56 | 2.34 | 0.901 |  | 36.15 | 34.91 | 1.52 | 0.533 |
| Day 8 | 40.89 | 38.59 | 2.34 | 0.321 |  | 39.17 | 36.83 | 1.35 | 0.238 |
| Day 14 | 44.93 | 41.33 | 2.34 | 0.124 |  | 41.75 | 39.36 | 1.39 | 0.215 |
| Day 18 | 44.93 | 41.45 | 2.45 | 0.156 |  | 43.09 | 38.47 | 1.36 | 0.020 |
|  | | | | |  |  |  |  |  |
| 24 h | | | | |  |  |  |  |  |
| Day 4 | 32.83 | 30.09 | 2.28 | 0.302 |  | 34.69 | 31.58 | 1.56 | 0.125 |
| Day 8 | 37.01 | 34.56 | 2.28 | 0.355 |  | 36.78 | 33.45 | 1.40 | 0.095 |
| Day 14 | 41.76 | 38.73 | 2.28 | 0.255 |  | 39.85 | 37.68 | 1.43 | 0.260 |
| Day 18 | 41.90 | 37.74 | 2.43 | 0.137 |  | 40.00 | 36.04 | 1.41 | 0.044 |

^1^Data are least squares means.

^2^Maximum value of the standard error of the means.

Table S9. Metabolic carbon dioxide (CO_2_) production [L/(d·BW^0.75^)] of lactating sows with litters fed high crude protein (HCP) and low crude protein (LCP) diet and exposed to thermal neutral and heat stress conditions^1^

| Item | Thermal neutral | | | |  | Heat stress | | | |
| --- | --- | --- | --- | --- | --- | --- | --- | --- | --- |
|  | HCP | LCP | SEM^2^ | *P*-value |  | HCP | LCP | SEM^2^ | *P*-value |
| Nighttime^4^ | | | | |  |  |  |  |  |
| Day 4 | 33.71 | 32.11 | 2.73 | 0.659 |  | 31.88 | 23.98 | 3.08 | 0.014 |
| Day 8 | 36.14 | 36.31 | 2.73 | 0.962 |  | 34.59 | 33.27 | 2.99 | 0.640 |
| Day 14 | 40.88 | 38.29 | 2.73 | 0.475 |  | 40.18 | 39.48 | 3.11 | 0.807 |
| Day 18 | 43.09 | 38.19 | 2.96 | 0.201 |  | 39.10 | 36.28 | 2.96 | 0.311 |
|  | | | | |  |  |  |  |  |
| Daytime | | | | |  |  |  |  |  |
| Day 4 | 33.91 | 32.29 | 2.70 | 0.534 |  | 35.07 | 34.40 | 1.55 | 0.747 |
| Day 8 | 38.66 | 37.20 | 2.70 | 0.574 |  | 37.90 | 34.35 | 1.39 | 0.083 |
| Day 14 | 42.32 | 39.67 | 2.70 | 0.313 |  | 40.29 | 37.39 | 1.42 | 0.146 |
| Day 18 | 42.32 | 40.04 | 2.82 | 0.406 |  | 41.57 | 36.21 | 1.40 | 0.009 |
|  | | | | |  |  |  |  |  |
| 24 h | | | | |  |  |  |  |  |
| Day 4 | 33.80 | 32.18 | 2.53 | 0.568 |  | 33.75 | 29.25 | 1.77 | 0.030 |
| Day 8 | 37.39 | 36.76 | 2.53 | 0.822 |  | 36.19 | 33.83 | 1.63 | 0.230 |
| Day 14 | 41.60 | 38.98 | 2.53 | 0.356 |  | 40.13 | 37.97 | 1.66 | 0.264 |
| Day 18 | 42.70 | 39.13 | 2.68 | 0.233 |  | 40.28 | 36.23 | 1.64 | 0.040 |

^1^Data are least squares means.

^2^Maximum value of the standard error of the means.

Table S10. Respiratory quotient (RQ) of lactating sows with litters fed high crude protein (HCP) and low crude protein (LCP) diet and exposed to thermal neutral and heat stress conditions^1^

| Item | Thermal neutral | | | |  | Heat stress | | | |
| --- | --- | --- | --- | --- | --- | --- | --- | --- | --- |
|  | HCP | LCP | SEM^2^ | *P*-value |  | HCP | LCP | SEM^2^ | *P*-value |
| Nighttime | | | | |  |  |  |  |  |
| Day 4 | 1.14 | 1.25 | 0.07 | 0.260 |  | 0.95 | 0.83 | 0.08 | 0.217 |
| Day 8 | 1.11 | 1.21 | 0.07 | 0.327 |  | 1.02 | 1.11 | 0.08 | 0.276 |
| Day 14 | 1.06 | 1.08 | 0.07 | 0.774 |  | 1.06 | 1.12 | 0.08 | 0.506 |
| Day 18 | 1.11 | 1.14 | 0.08 | 0.733 |  | 1.07 | 1.08 | 0.08 | 0.841 |
| Daytime | | | | |  |  |  |  |  |
| Day 4 | 0.98 | 0.93 | 0.02 | 0.124 |  | 0.96 | 1.00 | 0.02 | 0.131 |
| Day 8 | 0.94 | 0.98 | 0.02 | 0.178 |  | 0.97 | 0.94 | 0.02 | 0.209 |
| Day 14 | 0.94 | 0.96 | 0.02 | 0.444 |  | 0.96 | 0.95 | 0.02 | 0.464 |
| Day 18 | 0.95 | 0.96 | 0.02 | 0.735 |  | 0.97 | 0.94 | 0.02 | 0.303 |
| 24 h | | | | |  |  |  |  |  |
| Day 4 | 1.06 | 1.09 | 0.04 | 0.504 |  | 0.95 | 0.92 | 0.04 | 0.534 |
| Day 8 | 1.02 | 1.09 | 0.04 | 0.180 |  | 0.99 | 1.03 | 0.03 | 0.415 |
| Day 14 | 1.00 | 1.02 | 0.04 | 0.614 |  | 1.01 | 1.01 | 0.03 | 0.912 |
| Day 18 | 1.03 | 1.05 | 0.04 | 0.671 |  | 1.01 | 1.01 | 0.03 | 0.978 |

^1^Data are least squares means.

^2^Maximum value of the standard error of the means.

Table S11. Metabolic total heat production [kJ/(d·BW^0.75^)] during daytime of lactating sows with litters fed high crude protein (HCP) and low crude protein (LCP) diet and exposed to thermal neutral and heat stress conditions^1^

| Item | Thermal neutral | | | |  | Heat stress | | | |
| --- | --- | --- | --- | --- | --- | --- | --- | --- | --- |
|  | HCP | LCP | SEM^2^ | *P*-value |  | HCP | LCP | SEM^2^ | *P*-value |
| Day 4 |  |  |  |  |  |  |  |  |  |
| 07:00^3^ | 727.2 | 708.8 | 50.6 | 0.757 |  | 786.2 | 703.7 | 44.4 | 0.175 |
| 08:00 | 738.1 | 728.9 | 51.9 | 0.875 |  | 758.6 | 735.1 | 44.4 | 0.700 |
| 09:00 | 686.6 | 725.1 | 51.9 | 0.523 |  | 764.4 | 769.9 | 44.4 | 0.927 |
| 10:00 | 713.0 | 685.3 | 50.6 | 0.640 |  | 746.0 | 702.1 | 44.4 | 0.466 |
| 11:00 | 740.6 | 709.2 | 50.6 | 0.595 |  | 721.3 | 695.8 | 53.1 | 0.704 |
| 13:00 | 777.0 | 795.4 | 50.6 | 0.755 |  | 760.2 | 691.2 | 44.4 | 0.257 |
| 15:00 | 756.0 | 725.1 | 50.6 | 0.598 |  | 783.7 | 675.7 | 48.1 | 0.091 |
| 19:00 | 713.0 | 707.1 | 50.6 | 0.919 |  | 742.2 | 653.5 | 48.1 | 0.164 |
| Day 8 |  |  |  |  |  |  |  |  |  |
| 07:00^3^ | 808.3 | 741.0 | 59.4 | 0.362 |  | 817.1 | 684.9 | 41.4 | 0.029 |
| 08:00 | 874.5 | 848.5 | 59.4 | 0.724 |  | 866.5 | 785.3 | 41.4 | 0.174 |
| 09:00 | 816.7 | 749.8 | 59.4 | 0.363 |  | 841.0 | 774.9 | 41.4 | 0.267 |
| 10:00 | 861.9 | 797.5 | 59.4 | 0.380 |  | 803.3 | 725.9 | 41.4 | 0.194 |
| 11:00 | 851.0 | 824.2 | 59.4 | 0.716 |  | 819.2 | 769.9 | 41.4 | 0.406 |
| 13:00 | 880.3 | 848.9 | 59.4 | 0.672 |  | 831.4 | 717.1 | 41.4 | 0.058 |
| 15:00 | 912.9 | 864.4 | 59.4 | 0.510 |  | 905.4 | 766.1 | 41.4 | 0.022 |
| 19:00 | 857.7 | 810.0 | 59.4 | 0.519 |  | 817.6 | 813.0 | 41.4 | 0.940 |
| Day 14 |  |  |  |  |  |  |  |  |  |
| 07:00^3^ | 969.0 | 842.7 | 54.8 | 0.063 |  | 922.6 | 852.3 | 45.6 | 0.276 |
| 08:00 | 918.4 | 877.4 | 54.8 | 0.538 |  | 935.5 | 923.4 | 45.6 | 0.852 |
| 09:00 | 902.9 | 844.7 | 54.8 | 0.385 |  | 914.2 | 824.2 | 45.6 | 0.164 |
| 10:00 | 916.7 | 837.6 | 54.8 | 0.239 |  | 917.1 | 781.2 | 45.6 | 0.037 |
| 11:00 | 905.8 | 874.0 | 54.8 | 0.633 |  | 933.0 | 854.8 | 45.6 | 0.226 |
| 13:00 | 949.8 | 855.6 | 54.8 | 0.162 |  | 789.9 | 788.3 | 45.6 | 0.982 |
| 15:00 | 1,007.5 | 959.8 | 54.8 | 0.476 |  | 905.8 | 860.2 | 45.6 | 0.476 |
| 19:00 | 947.3 | 847.3 | 54.8 | 0.138 |  | 882.0 | 829.3 | 45.6 | 0.413 |
| Day 18 |  |  |  |  |  |  |  |  |  |
| 07:00^3^ | 949.8 | 876.1 | 63.6 | 0.345 |  | 928.0 | 866.1 | 45.2 | 0.337 |
| 08:00 | 998.3 | 896.2 | 63.6 | 0.192 |  | 951.0 | 810.4 | 45.2 | 0.033 |
| 09:00 | 894.5 | 830.5 | 63.6 | 0.411 |  | 860.6 | 790.4 | 45.2 | 0.279 |
| 10:00 | 867.8 | 780.3 | 63.6 | 0.263 |  | 853.5 | 728.4 | 45.2 | 0.056 |
| 11:00 | 901.2 | 851.0 | 63.6 | 0.517 |  | 916.7 | 766.9 | 45.2 | 0.023 |
| 13:00 | 944.7 | 869.0 | 63.6 | 0.333 |  | 1,024.2 | 811.3 | 49.0 | 0.004 |
| 15:00 | 1,001.2 | 918.8 | 63.6 | 0.291 |  | 969.4 | 787.0 | 49.0 | 0.021 |
| 19:00 | 935.5 | 838.5 | 63.6 | 0.214 |  | 888.3 | 837.6 | 49.0 | 0.486 |

^1^Data are least squares means.

^2^Maximum value of the standard error of the means.

^3^Total heat production before first morning meal.

Table S12. Metabolic oxygen (O_2_) consumption [L/(d·BW^0.75^)] during daytime of lactating sows with litters fed high crude protein (HCP) and low crude protein (LCP) diet and exposed to thermal neutral and heat stress conditions^1^

| Item | Thermal neutral | | | | |  | | Heat stress | | | | | |
| --- | --- | --- | --- | --- | --- | --- | --- | --- | --- | --- | --- | --- | --- |
|  | HCP | LCP | SEM^2^ | *P*-value |  | | HCP | | LCP | | SEM^2^ | | *P*-value |
| Day 4 |  |  |  |  | |  | |  | |  | |  |  |
| 07:00^3^ | 34.71 | 34.41 | 2.48 | 0.921 | |  | | 37.64 | | 33.64 | | 2.41 | 0.224 |
| 08:00 | 34.71 | 35.09 | 2.57 | 0.898 | |  | | 35.86 | | 34.08 | | 2.41 | 0.584 |
| 09:00 | 32.40 | 34.60 | 2.57 | 0.462 | |  | | 36.34 | | 36.09 | | 2.41 | 0.940 |
| 10:00 | 33.84 | 32.98 | 2.48 | 0.767 | |  | | 35.86 | | 33.64 | | 2.41 | 0.500 |
| 11:00 | 35.14 | 33.84 | 2.48 | 0.657 | |  | | 27.70 | | 32.92 | | 2.41 | 0.115 |
| 13:00 | 36.87 | 38.73 | 2.48 | 0.521 | |  | | 35.93 | | 32.49 | | 2.41 | 0.296 |
| 15:00 | 35.86 | 34.27 | 2.48 | 0.587 | |  | | 37.23 | | 32.20 | | 2.63 | 0.147 |
| 19:00 | 33.84 | 33.70 | 2.48 | 0.961 | |  | | 35.08 | | 31.34 | | 2.64 | 0.279 |
| Day 8 |  |  |  |  | |  | |  | |  | |  |  |
| 07:00^3^ | 39.46 | 34.71 | 2.92 | 0.194 | |  | | 39.13 | | 32.44 | | 2.05 | 0.026 |
| 08:00 | 41.90 | 40.61 | 2.92 | 0.722 | |  | | 41.01 | | 37.91 | | 2.05 | 0.294 |
| 09:00 | 38.88 | 35.14 | 2.92 | 0.305 | |  | | 40.14 | | 36.62 | | 2.05 | 0.233 |
| 10:00 | 41.47 | 38.16 | 2.92 | 0.364 | |  | | 37.83 | | 34.60 | | 2.05 | 0.273 |
| 11:00 | 40.46 | 39.60 | 2.92 | 0.812 | |  | | 38.85 | | 36.62 | | 2.05 | 0.449 |
| 13:00 | 41.76 | 40.61 | 2.92 | 0.751 | |  | | 39.56 | | 34.74 | | 2.05 | 0.105 |
| 15:00 | 43.78 | 41.76 | 2.92 | 0.580 | |  | | 43.02 | | 37.05 | | 2.05 | 0.046 |
| 19:00 | 40.75 | 38.59 | 2.92 | 0.553 | |  | | 39.13 | | 39.64 | | 2.05 | 0.863 |
| Day 14 |  |  |  |  | |  | |  | |  | |  |  |
| 07:00^3^ | 46.94 | 40.46 | 2.61 | 0.044 | |  | | 44.73 | | 40.87 | | 2.28 | 0.233 |
| 08:00 | 43.63 | 42.05 | 2.61 | 0.613 | |  | | 44.44 | | 44.18 | | 2.28 | 0.937 |
| 09:00 | 43.20 | 39.89 | 2.61 | 0.293 | |  | | 43.14 | | 39.29 | | 2.28 | 0.233 |
| 10:00 | 43.78 | 39.74 | 2.61 | 0.202 | |  | | 43.72 | | 37.41 | | 2.28 | 0.053 |
| 11:00 | 43.35 | 41.47 | 2.61 | 0.551 | |  | | 44.30 | | 40.73 | | 2.28 | 0.269 |
| 13:00 | 45.79 | 41.33 | 2.61 | 0.159 | |  | | 37.52 | | 37.64 | | 2.28 | 0.973 |
| 15:00 | 48.10 | 45.51 | 2.61 | 0.410 | |  | | 43.00 | | 41.30 | | 2.28 | 0.598 |
| 19:00 | 45.21 | 39.89 | 2.61 | 0.094 | |  | | 41.56 | | 39.43 | | 2.28 | 0.508 |
| Day 18 |  |  |  |  | |  | |  | |  | |  |  |
| 07:00^3^ | 46.22 | 42.25 | 3.16 | 0.308 | |  | | 44.45 | | 41.90 | | 2.28 | 0.430 |
| 08:00 | 48.24 | 42.65 | 3.16 | 0.153 | |  | | 45.32 | | 38.59 | | 2.28 | 0.041 |
| 09:00 | 42.34 | 39.30 | 3.16 | 0.435 | |  | | 40.56 | | 38.15 | | 2.28 | 0.456 |
| 10:00 | 41.04 | 37.13 | 3.16 | 0.315 | |  | | 40.43 | | 34.56 | | 2.28 | 0.073 |
| 11:00 | 42.77 | 40.74 | 3.16 | 0.601 | |  | | 43.45 | | 36.86 | | 2.28 | 0.045 |
| 13:00 | 45.07 | 41.45 | 3.16 | 0.352 | |  | | 49.23 | | 38.86 | | 2.46 | 0.006 |
| 15:00 | 48.10 | 43.79 | 3.16 | 0.269 | |  | | 46.17 | | 37.14 | | 2.47 | 0.023 |
| 19:00 | 44.35 | 40.11 | 3.16 | 0.276 | |  | | 42.16 | | 40.14 | | 2.47 | 0.579 |

^1^Data are least squares means.

^2^Maximum value of the standard error of the means.

^3^Prior to morning feeding at 07:00.

Table S13. Metabolic carbon dioxide (CO_2_) production [L/(d·BW^0.75^)] during daytime of lactating sows with litters fed high crude protein (HCP) and low crude protein (LCP) diet and exposed to thermal neutral and heat stress conditions^1^

| Item | Thermal neutral | | | |  | Heat stress | | | |
| --- | --- | --- | --- | --- | --- | --- | --- | --- | --- |
|  | HCP | LCP | SEM^2^ | *P*-value |  | HCP | LCP | SEM^2^ | *P*-value |
| Day 4 |  |  |  |  |  |  |  |  |  |
| 07:00^3^ | 32.83 | 30.24 | 2.44 | 0.375 |  | 35.73 | 31.81 | 2.42 | 0.236 |
| 08:00 | 34.85 | 31.89 | 2.49 | 0.320 |  | 36.00 | 36.86 | 2.42 | 0.794 |
| 09:00 | 31.82 | 32.55 | 2.44 | 0.804 |  | 35.82 | 37.58 | 2.42 | 0.594 |
| 10:00 | 33.26 | 30.67 | 2.44 | 0.375 |  | 33.70 | 31.24 | 2.42 | 0.454 |
| 11:00 | 34.56 | 32.55 | 2.44 | 0.489 |  | 30.83 | 32.82 | 2.42 | 0.544 |
| 13:00 | 36.57 | 33.98 | 2.44 | 0.375 |  | 35.13 | 33.69 | 2.42 | 0.661 |
| 15:00 | 34.56 | 33.84 | 2.44 | 0.804 |  | 36.08 | 31.67 | 2.65 | 0.204 |
| 19:00 | 32.69 | 32.25 | 2.44 | 0.882 |  | 34.67 | 29.65 | 2.66 | 0.150 |
| Day 8 |  |  |  |  |  |  |  |  |  |
| 07:00^3^ | 34.85 | 35.86 | 2.72 | 0.764 |  | 36.68 | 31.48 | 2.04 | 0.080 |
| 08:00 | 39.31 | 38.45 | 2.72 | 0.797 |  | 40.42 | 34.21 | 2.04 | 0.038 |
| 09:00 | 37.30 | 36.57 | 2.72 | 0.830 |  | 38.55 | 37.24 | 2.04 | 0.655 |
| 10:00 | 38.59 | 36.29 | 2.72 | 0.493 |  | 38.12 | 33.50 | 2.04 | 0.118 |
| 11:00 | 38.73 | 37.15 | 2.72 | 0.637 |  | 37.97 | 34.79 | 2.04 | 0.279 |
| 13:00 | 40.46 | 38.59 | 2.72 | 0.578 |  | 37.69 | 31.19 | 2.04 | 0.030 |
| 15:00 | 40.89 | 37.87 | 2.72 | 0.370 |  | 41.58 | 33.50 | 2.04 | 0.008 |
| 19:00 | 39.60 | 36.57 | 2.72 | 0.370 |  | 36.82 | 34.65 | 2.04 | 0.459 |
| Day 14 |  |  |  |  |  |  |  |  |  |
| 07:00^3^ | 42.19 | 37.58 | 2.66 | 0.164 |  | 40.06 | 38.07 | 2.02 | 0.483 |
| 08:00 | 41.90 | 39.46 | 2.66 | 0.455 |  | 43.37 | 41.24 | 2.02 | 0.452 |
| 09:00 | 41.04 | 38.88 | 2.66 | 0.509 |  | 43.08 | 38.07 | 2.02 | 0.081 |
| 10:00 | 41.33 | 39.17 | 2.66 | 0.509 |  | 41.50 | 35.62 | 2.02 | 0.042 |
| 11:00 | 41.19 | 39.89 | 2.66 | 0.692 |  | 42.51 | 39.08 | 2.02 | 0.229 |
| 13:00 | 42.19 | 37.73 | 2.66 | 0.178 |  | 36.46 | 34.90 | 2.02 | 0.582 |
| 15:00 | 45.79 | 44.50 | 2.66 | 0.692 |  | 41.93 | 38.21 | 2.02 | 0.193 |
| 19:00 | 43.05 | 40.46 | 2.66 | 0.429 |  | 40.92 | 38.07 | 2.02 | 0.316 |
| Day 18 |  |  |  |  |  |  |  |  |  |
| 07:00^3^ | 40.32 | 38.37 | 2.93 | 0.586 |  | 41.38 | 37.99 | 2.00 | 0.237 |
| 08:00 | 43.78 | 41.08 | 2.93 | 0.452 |  | 43.68 | 36.98 | 2.00 | 0.022 |
| 09:00 | 41.62 | 38.98 | 2.93 | 0.461 |  | 40.37 | 33.96 | 2.00 | 0.028 |
| 10:00 | 40.61 | 36.10 | 2.93 | 0.211 |  | 39.79 | 33.82 | 2.00 | 0.040 |
| 11:00 | 41.90 | 39.35 | 2.93 | 0.476 |  | 42.38 | 34.39 | 2.00 | 0.007 |
| 13:00 | 43.05 | 40.12 | 2.93 | 0.413 |  | 44.95 | 37.17 | 2.15 | 0.017 |
| 15:00 | 44.35 | 41.71 | 2.93 | 0.461 |  | 44.49 | 36.79 | 2.16 | 0.018 |
| 19:00 | 43.05 | 37.90 | 2.93 | 0.154 |  | 41.28 | 38.46 | 2.16 | 0.374 |

^1^Data are least squares means.

^2^Maximum value of the standard error of the means.

^3^Prior to morning feeding at 07:00.

Table S14. Respiratory quotient (RQ) during daytime of lactating sows with litters fed high crude protein (HCP) and low crude protein (LCP) diet and exposed to thermal neutral and heat stress conditions^1^

| Item | Thermal neutral | | | |  | Heat stress | | | |
| --- | --- | --- | --- | --- | --- | --- | --- | --- | --- |
|  | HCP | LCP | SEM^2^ | *P*-value |  | HCP | LCP | SEM^2^ | *P*-value |
| Day 4 |  |  |  |  |  |  |  |  |  |
| 07:00^3^ | 0.95 | 0.88 | 0.04 | 0.180 |  | 0.95 | 0.94 | 0.04 | 0.906 |
| 08:00 | 1.01 | 0.91 | 0.04 | 0.071 |  | 0.99 | 1.07 | 0.04 | 0.135 |
| 09:00 | 0.98 | 0.96 | 0.04 | 0.633 |  | 0.98 | 1.04 | 0.04 | 0.265 |
| 10:00 | 0.99 | 0.94 | 0.04 | 0.394 |  | 0.94 | 0.95 | 0.04 | 0.812 |
| 11:00 | 0.98 | 0.97 | 0.04 | 0.816 |  | 0.98 | 1.00 | 0.05 | 0.723 |
| 13:00 | 0.99 | 0.88 | 0.04 | 0.037 |  | 0.98 | 1.04 | 0.04 | 0.253 |
| 15:00 | 0.97 | 0.99 | 0.04 | 0.725 |  | 0.95 | 0.98 | 0.04 | 0.521 |
| 19:00 | 0.97 | 0.95 | 0.04 | 0.752 |  | 0.97 | 0.94 | 0.04 | 0.613 |
| Day 8 |  |  |  |  |  |  |  |  |  |
| 07:00^3^ | 0.87 | 1.10 | 0.05 | 0.003 |  | 0.95 | 0.98 | 0.03 | 0.484 |
| 08:00 | 0.93 | 0.94 | 0.05 | 0.897 |  | 0.99 | 0.90 | 0.03 | 0.035 |
| 09:00 | 0.96 | 1.10 | 0.05 | 0.056 |  | 0.96 | 1.01 | 0.03 | 0.247 |
| 10:00 | 0.93 | 0.95 | 0.05 | 0.820 |  | 1.00 | 0.96 | 0.03 | 0.308 |
| 11:00 | 0.96 | 0.94 | 0.05 | 0.838 |  | 0.98 | 0.94 | 0.03 | 0.376 |
| 13:00 | 0.97 | 0.96 | 0.05 | 0.891 |  | 0.95 | 0.90 | 0.03 | 0.177 |
| 15:00 | 0.93 | 0.90 | 0.05 | 0.700 |  | 0.96 | 0.91 | 0.03 | 0.188 |
| 19:00 | 0.97 | 0.94 | 0.05 | 0.727 |  | 0.95 | 0.90 | 0.03 | 0.213 |
| Day 14 |  |  |  |  |  |  |  |  |  |
| 07:00^3^ | 0.90 | 0.93 | 0.03 | 0.347 |  | 0.91 | 0.93 | 0.02 | 0.524 |
| 08:00 | 0.95 | 0.94 | 0.03 | 0.641 |  | 0.98 | 0.93 | 0.02 | 0.150 |
| 09:00 | 0.95 | 0.97 | 0.03 | 0.595 |  | 1.00 | 0.97 | 0.02 | 0.314 |
| 10:00 | 0.94 | 0.99 | 0.03 | 0.166 |  | 0.95 | 0.96 | 0.02 | 0.831 |
| 11:00 | 0.95 | 0.96 | 0.03 | 0.915 |  | 0.95 | 0.97 | 0.02 | 0.652 |
| 13:00 | 0.93 | 0.93 | 0.03 | 0.981 |  | 0.97 | 0.93 | 0.02 | 0.198 |
| 15:00 | 0.96 | 0.98 | 0.03 | 0.602 |  | 0.97 | 0.93 | 0.02 | 0.157 |
| 19:00 | 0.95 | 1.02 | 0.03 | 0.061 |  | 0.98 | 0.96 | 0.02 | 0.626 |
| Day 18 |  |  |  |  |  |  |  |  |  |
| 07:00^3^ | 0.92 | 0.91 | 0.04 | 0.977 |  | 0.93 | 0.91 | 0.03 | 0.542 |
| 08:00 | 0.91 | 0.98 | 0.04 | 0.153 |  | 0.97 | 0.96 | 0.03 | 0.800 |
| 09:00 | 0.98 | 0.99 | 0.04 | 0.866 |  | 0.99 | 0.90 | 0.03 | 0.017 |
| 10:00 | 0.98 | 0.97 | 0.04 | 0.777 |  | 0.99 | 0.98 | 0.03 | 0.927 |
| 11:00 | 0.98 | 0.97 | 0.04 | 0.796 |  | 0.98 | 0.94 | 0.03 | 0.265 |
| 13:00 | 0.95 | 0.98 | 0.04 | 0.507 |  | 0.92 | 0.94 | 0.03 | 0.642 |
| 15:00 | 0.92 | 0.95 | 0.04 | 0.624 |  | 0.96 | 0.98 | 0.03 | 0.589 |
| 19:00 | 0.98 | 0.93 | 0.04 | 0.334 |  | 0.99 | 0.95 | 0.03 | 0.419 |

^1^Data are least squares means.

^2^Maximum value of the standard error of the means.

^3^Prior to morning feeding at 07:00.

Table S15. Metabolic carbon dioxide (CO_2_) production, oxygen (O_2_) consumption, total heat production (THP) and respiratory quotient (RQ) of piglets from sows fed high crude protein (HCP) and low crude protein (LCP) diet and exposed to thermal neutral and heat stress conditions^1^

| Item | BW, kg^2^ | CO_2_, L/(d·BW^0.75^) | O_2_, L/(d·BW^0.75^) | THP, kJ/(d·BW^0.75^) | RQ |
| --- | --- | --- | --- | --- | --- |
| Heat stress |  |  |  |  |  |
| HCP |  |  |  |  |  |
| Day 4 | 2.20 | 31.28 | 41.30 | 824.2 | 0.76 |
| Day 8 (9) | 3.46 | 40.35 | 41.73 | 878.1 | 0.97 |
| Day 14 (15) | 5.47 | 41.99 | 47.43 | 977.3 | 0.89 |
| Day 18 (19) | 6.13 | 41.21 | 47.35 | 972.5 | 0.87 |
| LCP |  |  |  |  |  |
| Day 4 | 2.10 | 24.36 | 34.39 | 678.0 | 0.71 |
| Day 8 (9) | 4.50 | 33.96 | 39.66 | 811.9 | 0.86 |
| Day 14 (15) | 5.68 | 34.91 | 45.79 | 915.8 | 0.76 |
| Day 18 (17) | 5.65 | 47.87 | 56.42 | 1,152.5 | 0.85 |
| Thermal neutral |  |  |  |  |  |
| HCP |  |  |  |  |  |
| Day 4 | 1.75 | 34.56 | 38.88 | 802.3 | 0.89 |
| Day 8 (9) | 3.47 | 35.42 | 43.20 | 869.6 | 0.82 |
| Day 14 (13) | 5.17 | 40.61 | 44.93 | 910.8 | 0.90 |
| Day 18 (17) | 6.10 | 43.20 | 49.25 | 1,007.2 | 0.88 |
| LCP |  |  |  |  |  |
| Day 4 (3) | 2.19 | 31.97 | 39.74 | 803.3 | 0.80 |
| Day 8 (9) | 3.42 | 38.02 | 41.47 | 856.5 | 0.92 |
| Day 14 (15) | 5.18 | 36.29 | 48.38 | 962.0 | 0.75 |
| Day 18 | 7.20 | 41.47 | 47.52 | 1,032.3 | 0.87 |

^1^Acual day of lactation is shown in parentheses.

^2^BW: body weight.
